# Seq: A High-Performance Language for Bioinformatics

**DOI:** 10.1145/3360551

**Published:** 2019-10-10

**Authors:** ARIYA SHAJII, IBRAHIM NUMANAGIĆ, RIYADH BAGHDADI, BONNIE BERGER, SAMAN AMARASINGHE, MIT CSAIL, USA

**Affiliations:** MIT CSAIL, 77 Massachusetts Ave, Cambridge, MA, 02139, USA.

**Keywords:** computational biology, bioinformatics, programming language, domain-specific language, Python, optimization

## Abstract

The scope and scale of biological data are increasing at an exponential rate, as technologies like next-generation sequencing are becoming radically cheaper and more prevalent. Over the last two decades, the cost of sequencing a genome has dropped from $100 million to nearly $100—a factor of over 10^6^—and the amount of data to be analyzed has increased proportionally. Yet, as Moore’s Law continues to slow, computational biologists can no longer rely on computing hardware to compensate for the ever-increasing size of biological datasets. In a field where many researchers are primarily focused on biological analysis over computational optimization, the unfortunate solution to this problem is often to simply buy larger and faster machines.

Here, we introduce Seq, the first language tailored specifically to bioinformatics, which marries the ease and productivity of Python with C-like performance. Seq starts with a subset of Python—and is in many cases a drop-in replacement—yet also incorporates novel bioinformatics- and computational genomics-oriented data types, language constructs and optimizations. Seq enables users to write high-level, Pythonic code without having to worry about low-level or domain-specific optimizations, and allows for the seamless expression of the algorithms, idioms and patterns found in many genomics or bioinformatics applications. We evaluated Seq on several standard computational genomics tasks like reverse complementation, *k*-mer manipulation, sequence pattern matching and large genomic index queries. On equivalent CPython code, Seq attains a performance improvement of up to two orders of magnitude, and a 160× improvement once domain-specific language features and optimizations are used. With parallelism, we demonstrate up to a 650× improvement. Compared to optimized C++ code, which is already difficult for most biologists to produce, Seq frequently attains up to a 2× improvement, and with shorter, cleaner code. Thus, Seq opens the door to an age of democratization of highly-optimized bioinformatics software.

## 1 INTRODUCTION

DNA sequencing technologies have revolutionized life sciences and clinical medicine [Mardis 2017]. Today, state-of-the-art cancer treatment involves sequencing a tumor genome for diagnosis and ensuing care [Kamps et al. 2017]. Improvements in sequencing hardware have drastically reduced the laboratory cost of sequencing; thus, the biggest bottleneck and cost today in the sequence analysis pipeline is computational data analysis [Muir et al. 2016]. Indeed, reductions in sequencing costs over the past two decades have radically outpaced both Moore’s and Kryder’s Laws. Sequencing a genome in 2008 cost more than $10 million, but over the past several years the cost has fallen below the celebrated $1,000 mark, and is expected to soon surpass $100 per genome [Hayden 2014]. Unfortunately, the computational data analysis cost inherent in sequencing pipelines has not improved at the same rate, and in 2011 surpassed the cost of sequencing [Sboner et al. 2011]. In fact, it is expected to soon be cheaper to simply re-sequence an individual than to even store their raw sequencing data, let alone analyze it [Weymann et al. 2017]. By the same token, the number and size of sequencing datasets continue to grow exponentially, which will require better algorithms and software especially as the gap between Moore’s Law and sequencing data growth continues to widen.

Recent advances in next-generation sequencing (NGS) technologies are continuing this revolution, and providing a means to study various biological processes through a genomic lens. Because of its novel capabilities and vast scale, NGS has even bigger computational needs, as terabytes of data need to be processed and analyzed with the aid of various novel computational methods and tools [Mardis 2017]. These tools (e.g. [Li et al. 2009a], [Li and Durbin 2009], [Zaharia et al. 2011], [McKenna et al. 2010], [Yorukoglu et al. 2016], [Shajii et al. 2018]) are used on a daily basis in research laboratories and have fueled major discoveries such as establishing mutation-disease links [Manolio et al. 2008] and detecting recent segmental duplications in the genome [Bailey et al.2001].

Despite these advances, however, many contemporary genomic pipelines cannot scale with the ever-increasing deluge of sequencing data, which has necessitated impractical and expensive *ad hoc* solutions such as frequent hardware upgrades and constant (re-)implementation of underlying software. Many promising methods are also too difficult to use and replicate because they are often manually tuned for a single dataset, further fueling the recent replication crisis [Baker 2016; Peng 2011]. Finally, many tools are not maintained due to a lack of personnel, expertise and funds, which has led to many abandoned code repositories that cannot easily be modified to suit researchers’ needs, although being vastly superior to the well-maintained alternatives in theory.

The root cause of these problems lies in the general-purpose languages that are used for bioinformatics software development. The most popular programming languages, Python, R, and occasionally C++, are not designed to efficiently handle and optimize for sequencing data workflows. Even so, researchers often use high-level languages like Python or R to analyze NGS data since they allow for the quick and easy expression of high-level ideas, despite a steep performance penalty. Alternatively, a researcher may manually implement low-level optimizations in a language like C. However, doing so requires a considerable time investment and often results in hard-to-maintain codebases ridden with subtle bugs and tied to a particular architecture, especially in a field like computational biology where many researchers are not software engineers by trade. These issues are further exacerbated as the field shifts towards the use of third-generation portable sequencers that are powered by resource-limited devices [Lu et al. 2016], which warrant entirely different software designs and optimizations.

In this paper, we introduce *Seq*^1^, a domain-specific language (DSL) and compiler designed to provide productivity *and* high performance for computational biology. Seq is a subset of Python, and therefore provides Python-level productivity; yet, the compiler can generate efficient code because the language is statically-typed with compile-time support for Python’s duck typing. Seq provides data types tailored to computational genomics and uses domain-specific information to optimize code. Our DSL allows computational biology experts to quickly prototype and experiment with new algorithms as they would in Python, without imposing the burden of learning a new language. Further, Seq is designed to hide all low-level, complex code optimizations from the end user. Unlike libraries, the Seq compiler can perform optimizations such as operator fusion and pipeline transformations, which we demonstrate to have a substantial benefit.

This paper makes the following contributions:

- We introduce Seq, the first domain-specific language and compiler for computational biology.
- We introduce novel genomics-specific data types (e.g. sequence and *k*-mer types) and operators (e.g. for reverse complementation, *k*-merization, etc.), further augmented with additional language constructs such as compiler-optimized pipelines and genomic matching, to both simplify the algorithmic descriptions of complex problems and to enable domain-specific optimizations.
- We design a statically-typed subset of Python tailored for bioinformatics applications, which operates entirely without expensive runtime type information and provides performance comparable to (and in many cases better than) C’s.
- We demonstrate how to use parallelism, prefetching, pipelining and coroutines to drastically improve the performance of Seq.
- We show that many important and widely-used NGS algorithms can be made up to 160× faster than their Python counterparts as well as 2× faster than the existing hand-optimized C++ implementations.
- We provide an implementation of the compiler and standard library.

The rest of the paper is organized as follows. Section 2 provides a primer on computational genomics. Section 3 gives an example of the Seq language, followed by a description of the language design and implementation in Section 4 and domain-specific optimizations in Section 5. Section 6 evaluates the language, Section 7 describes related work, and the paper concludes in Section 8.

## 2 A PRIMER ON COMPUTATIONAL GENOMICS

**Fig. 1.**
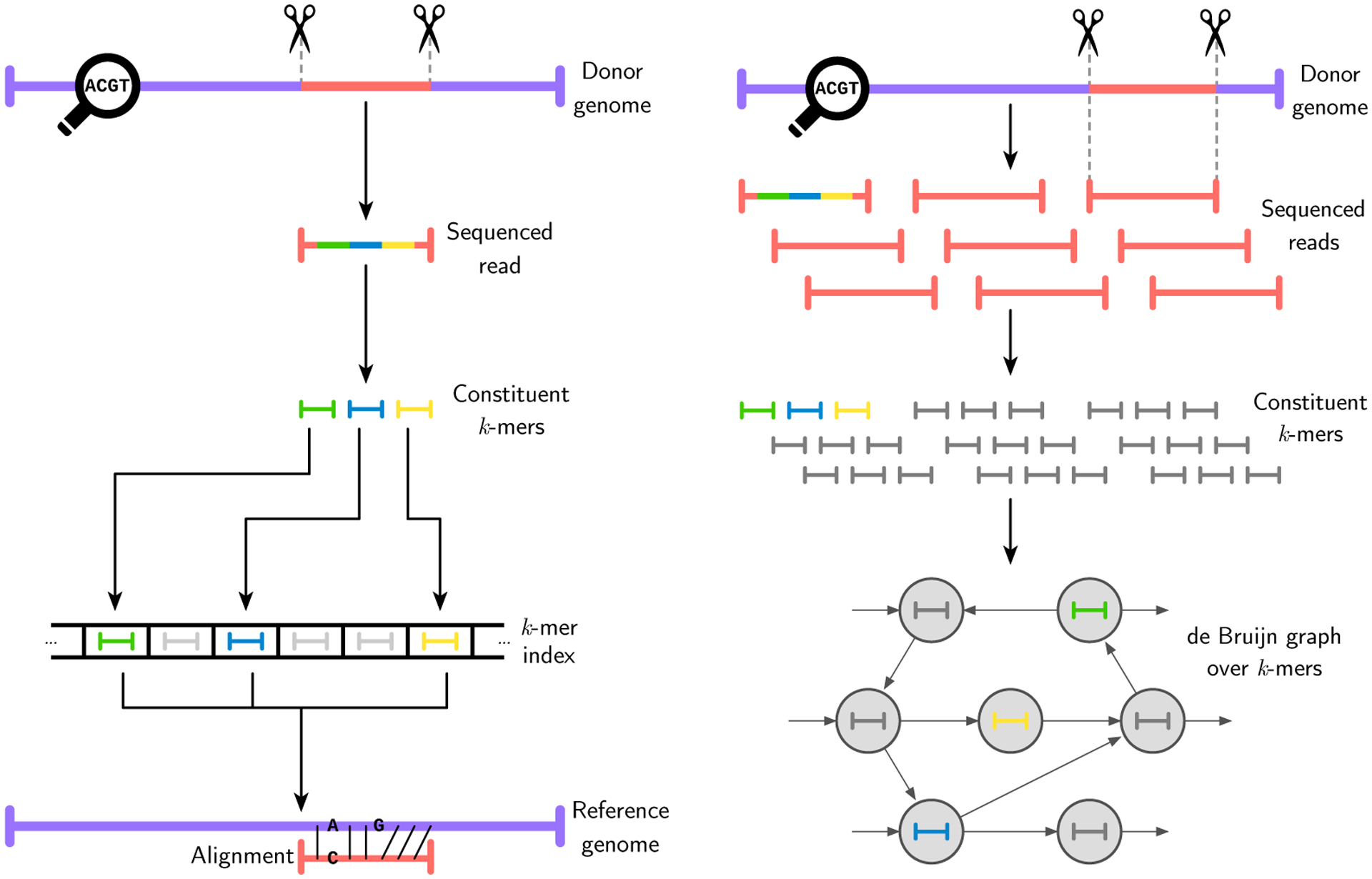
Visualizations of two standard computational genomics applications. (a) Overview of the alignment process for sequencing data. A sequencing machine produces a read: a roughly 100 base pair DNA sequence randomly sampled from the donor’s genome. Most alignment algorithms then split this read into *k*-mers—fixed length-*k* subsequences—and query these *k*-mers in an index of *k*-mers from the reference genome to determine candidate alignment positions. Finally, full dynamic programming alignment (typically via an adapted Smith-Waterman algorithm) is carried out to produce the final alignment. (b) Overview of de novo genome assembly from sequencing data. Sequenced reads are partitioned into constituent *k*-mers, which are then taken to be nodes in a de Bruijn graph whose edges represent (*k* − 1)length overlaps. Other formulations use (*k* − 1)-mers (two for each original *k*-mer) as nodes with the original *k*-mers represented by the edges. The assembled sequence corresponds to an Eulerian path on this graph.

The fundamental data type in computational genomics is the *sequence*, which is conceptually a string over Σ = {A, C, G, T}, representing the four nucleotides (also called *bases*) that comprise DNA. Sequences come in several different forms, with varying properties such as length, error profile and metadata. For example, *genome sequencing*—a process that determines the DNA content of a given biological sample—typically produces *reads*: DNA sequences roughly 100 bases in length, with a substitution error rate less than 1% and metadata consisting of a unique identifier and a string of *quality scores*, indicating the sequencing machine’s confidence in each reported base of the read. Reads are often analyzed in the context of a *reference genome*, a much longer (in the case of human, 3 gigabase-length) sequence that represents the consensus sequence of an organism’s genome in its entirety. A standard first step in nearly any sequence analysis pipeline is *sequence alignment*, which is the process of identifying the position in the reference sequence to which a particular read aligns with the smallest edit distance (although many different formulations of this problem exist, such as finding *all* alignments under a given edit distance threshold). To this end, reads are typically first split into fixed length-*k* contiguous subsequences called *k-mers*, which are then queried in an index of *k*-mers from the reference to guide the alignment process, as shown in Figure 1a. The index itself is an abstract data type that maps *k*-mers to positions (also called *loci*) in the reference at which they appear, and is often implemented in practice as a hash table or FM-index [Ferragina and Manzini 2004; Li and Homer 2010].

Due to the large memory footprints of these structures (roughly 5 gigabytes for optimized FM-indices and tens of gigabytes for hash tables) given the size of the genome, coupled with their poor cache performance, many alignment algorithms spend a significant fraction of their time time stalled on memory accesses; the fraction of stalled cycles in these applications can be over 70% depending on the input dataset [Appuswamy et al. 2018]. Once a candidate locus is found via the index (and possibly after several filtering steps), a full dynamic programming alignment is performed, usually via a variant of the Smith-Waterman algorithm. Because dynamic programming alignment is a key kernel in nearly all alignment algorithms, there has been substantial research into designing hand-optimized implementations that exploit SIMD vectorization for better performance [Farrar 2006; Suzuki and Kasahara 2018; Šošić and Šikić 2017]. One additional complication in sequence alignment is that, while half of all reads will align in the so-called *forward direction* (i.e. without modification), the other half will only align in the *reverse* direction, meaning the read must be *reverse complemented* before alignment. Reverse complementation of a sequence is an operation where the sequence is reversed, and A-bases are swapped with T-bases while C-bases are swapped with G-bases (and vice versa). The fact that half the reads are reverse complemented with respect to the reference genome is a biproduct of the double-stranded nature of DNA, and ultimately leads to reverse complementation being a very common operation that is done on sequences.

Alongside alignment, another common application in computational genomics is *de novo* assembly, where the reads are used to “reconstruct” the donor genome, in the absence of a predefined reference sequence. While several approaches to this problem exist, perhaps the most common is to again partition the reads into *k*-mers, build a de Bruijn graph whose vertices are these *k*-mers with edges indicating that a given *k*-mer overlaps with another, and finally to find an Eulerian path through this graph, which would encode the assembled sequence [Khan et al. 2018]. An overview of this process can be seen in Figure 1b. As in alignment, there are several additional steps involved in practice, such as counting and filtering *k*-mers, as well as error correction (as assembly is more sensitive to errors than alignment) [Simpson and Durbin 2012].

Looking further downstream in the genomic analysis pipeline, computational biologists employ a slew of techniques to handle the problems at hand. However, virtually any downstream model or algorithm, regardless of its domain (machine learning, graph algorithms, etc.), is built on top of the sequence manipulation building blocks described above. For example, structural variation detection (the discovery of novel genomic rearrangements) starts by analyzing read alignment irregularities to detect potential breakpoints of a rearrangement, and proceeds by correcting those alignments via more advanced read alignment schemes that utilize *k*-mers and FM-indices. Many other problems, such as mutation calling, gene copy number variation detection, genome-wide association studies and cancer driver identification, proceed in a similar fashion. Thus, the common threads between alignment, assembly and many other applications in the genomics domain are the data types used (i.e. various types of sequences like reads, reference or fixed-length *k*-mers) and the low-level operations performed on them (i.e. some form of matching, indexing, splitting sequences into subsequences or *k*-mers, reverse complementation, etc.). However, these operations are often embedded in vastly different higher-level algorithms; compare, for example, the dynamic programming involved in alignment and the de Bruijn graph path finding involved in assembly. For this reason, we chose to expose these genomics-specific types and operations in a comparatively lower-level language than many other DSLs, as we discuss in detail below.

## 3 SEQUENCE *k*-MERIZATION AND SEEDING — AN EXAMPLE

Seq provides built-in language-level facilities for seamlessly expressing many of the types and design patterns found in genomics applications. As an example, consider reading a set of sequencing reads from a FASTQ file (a standard format for storing reads) and querying each read’s constituent *k*-mers in a genomic index. This process is commonly referred to as *seeding*, and is the first step in nearly any sequence alignment algorithm [Li and Homer 2010].

**Fig. 2.**
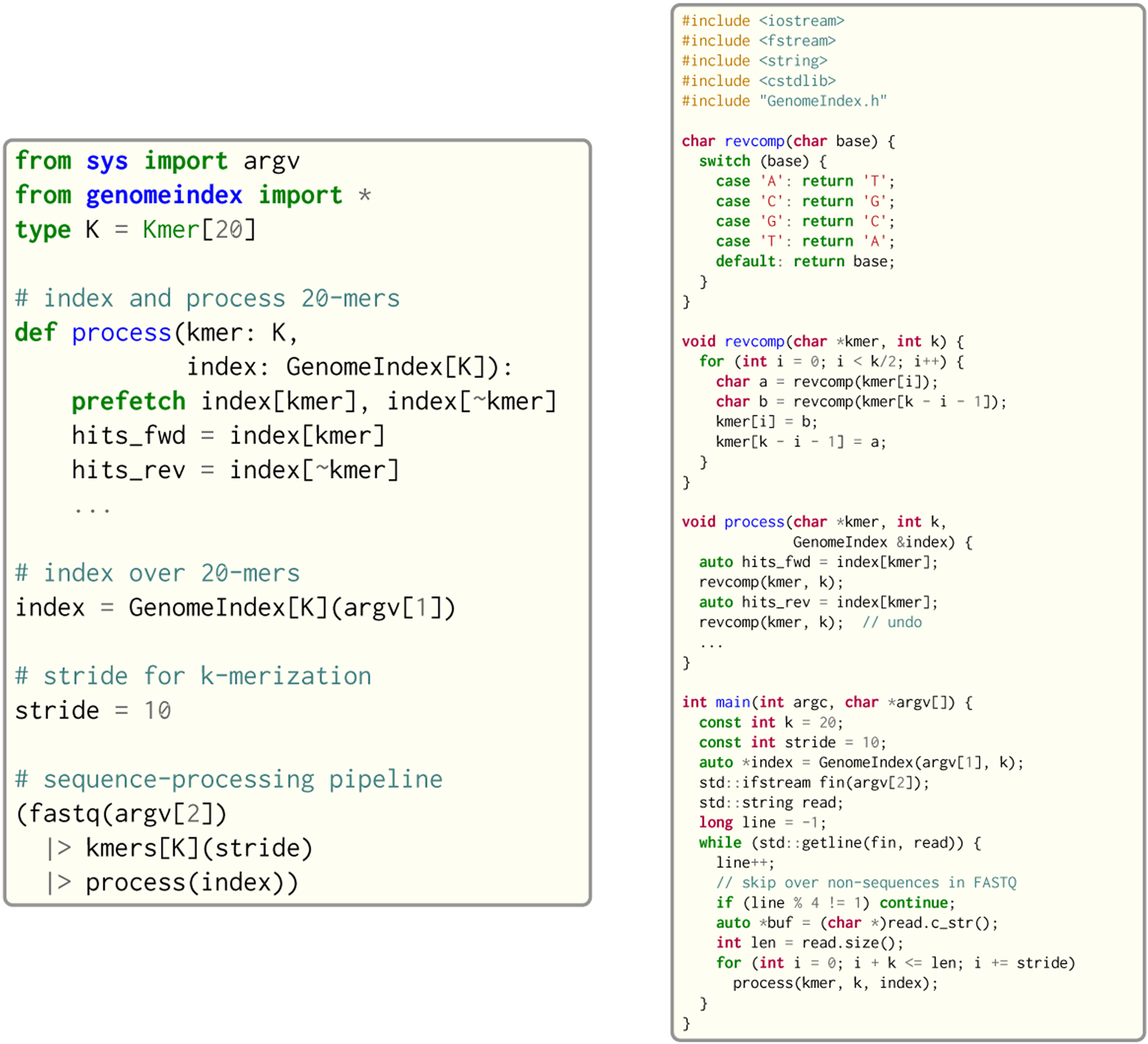
Example *k*-merization and seeding application in Seq and C++.

An implementation of 20-mer seeding in Seq is shown in Figure 2. Seq uses the familiar syntax of Python, but incorporates several genomics-specific features and optimizations. *k*-mer types like Kmer[20] (which represents a *k*-mer with 20 bases), for example, allow for easy *k*-merization (the process of splitting a sequence into *k*-mers, done using kmers in Seq) and reverse complementation (using ~kmer). Similarly, pipelining—a natural model for thinking about processing reads—is easily expressible in Seq, where a user can define pipelines via the |> operator as shown in the figure. Seq can also speed up expensive index queries via pipeline transformations that allow for effective software prefetching (prefetch keyword). Compare this Seq implementation to the C++ implementation also shown in Figure 2, which includes extensive boilerplate code for reverse complementation and FASTQ iteration, and cannot perform the domain-specific pipeline or encoding optimizations made by the Seq compiler, which in practice we find to attain upwards of 1.5–2× speedups over optimized C++ implementations.

## 4 LANGUAGE DESIGN & IMPLEMENTATION

A critical barrier to any new language’s success in a particular field is its initial adoption, as most potential users already have a set of languages, environments and packages with which they are comfortable. This is particularly true in bioinformatics, where many researchers are biologists first and programmers second. For this reason, the Seq language borrows the syntax and semantics of Python—one of the most widely-used languages in bioinformatics—and adds several genomics-oriented language features and constructs. Indeed, most of the preexisting Python code that is used within the genomics community will compile and run without modification in Seq, ultimately allowing the user to attain the performance of C/C++ with the programming ease of Python.

To achieve this, we designed a compiler with a static type system. It performs Python-style duck typing and runtime type checking at compile time, completely eliminating the substantial runtime overhead imposed by the reference Python implementation, CPython, and most other Python implementations alike. Unlike these, we reimplemented all of Python’s language features and built-in facilities from the ground up, completely independent of the CPython runtime. The Seq compiler uses an LLVM [Lattner and Adve 2004] backend, and in general uses LLVM as a framework for performing general-purpose optimizations. Seq programs additionally use a lightweight (<200 LOC) runtime library for I/O and memory allocation; for the latter, CPython’s reference counting is replaced with the Boehm garbage collector [Boehm and Weiser 1988], a widely-used conservative GC that is a drop-in replacement for malloc.

(“Python” is henceforth used as a synonym for “CPython” unless otherwise specified.)

### 4.1 Building a Statically-Typed Python

Python is an interpreted language, and does not check for type consistency until necessary during runtime—even then, only the existence of methods required by a given program is checked, an approach commonly referred to as *duck typing*. This simple and clean design, together with a well-thought-out syntax, enables rapid prototyping and a great deal of flexibility without imposing artificial language design constraints, which is partly what has made Python popular in many different domains, bioinformatics notwithstanding.

However, this dynamism comes with a hefty price in terms of performance, as almost any method invocation or variable reference requires expensive dictionary lookups during runtime. Furthermore, the lack of type annotations and the dynamic nature of objects necessitates delaying type checks until a given object is actually used, which can sometimes be *days* after the Python script was initially run in the case of long-running programs (a common problem in bioinformatics, where many scripts take a long time to complete due to ever-growing input datasets, whereby rapid initial development is paid for by a slow debugging cycle). Moreover, this lazy approach to typing requires the developer to include numerous manual type checks and large test suites to ensure type soundness during execution. Python versions 3.6 and later attempt to mitigate this problem with the optional mypy type checker, which adds support for type annotations with ahead-of-time type checks to the core language (and whose syntax we adopted for consistency). However, mypy must still interoperate with the Python runtime, and as such could leave some types ambiguous (e.g. as Any), which does not map easily to LLVM IR—the backbone of Seq’s optimization framework. PyPy, on the other hand, uses a restricted subset of Python called “RPython” which can be statically typed, but again does not fit our purposes as it performs type deduction at runtime and allows arbitrary non-RPython code to be mixed in. By contrast, the Seq compiler has a complete view of *all* types at compilation time, which it uses to avoid all runtime overhead.

In most domains, a lack of performance is a fair price to pay for Python’s ease and expressibility as compared to the alternatives. However, this is significantly problematic in the context of computational biology, where an average dataset is on the order of hundreds of gigabytes in size, and where even a simple loop construct incurs enough overhead to render Python code hundreds of times slower than its C counterpart. Highly optimized Python implementations, such as PyPy or Numba, do not sufficiently address these problems as they are either bound to the same constraints as the original (CPython) implementation (i.e. dynamic runtime and lazy duck typing), or limited in scope solely to numerical types.

Despite its array of dynamic and runtime-oriented features, the full flexibility provided by Python is not commonly used in many domains. While type flexibility and dynamic object/type modifications are, for example, crucial for rapid web development, they are almost completely absent in high-performance scientific applications, and arguably even slow down the development cycle of such applications. For these reasons, we designed a strongly-typed alternative to Python’s runtime, which captures the subset of its dynamic features that is commonly used in the field of computational biology, and that can be resolved *at compile time*. In doing so, we trade some largely unneeded dynamism for greatly improved performance, which by contrast *is* gravely needed in the field.

#### Basic Types.

**Fig. 3.**
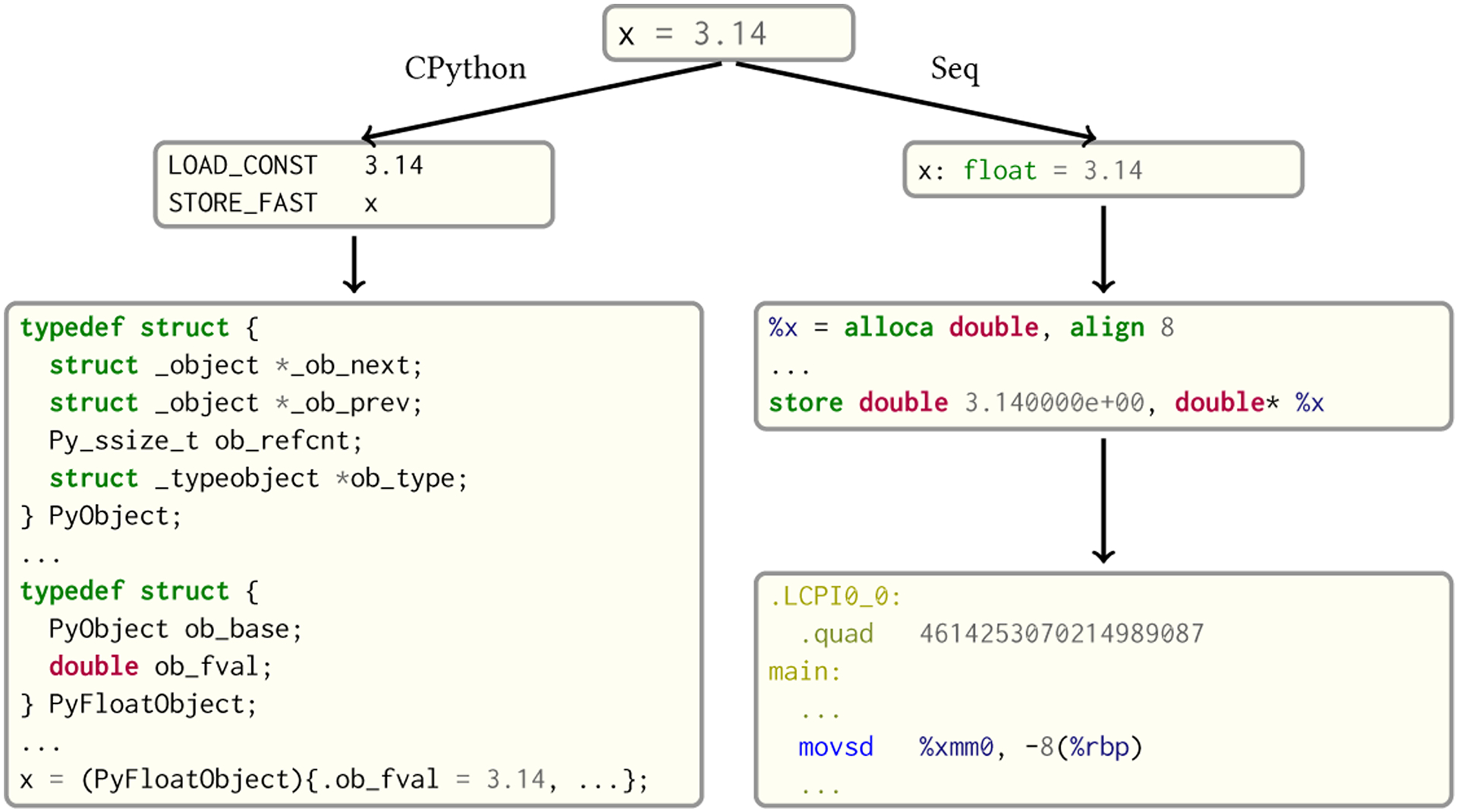
Seq versus CPython during compilation and execution of a simple float assignment. CPython compiles to bytecode that omits all type information, and instead relies on runtime type information by virtue of metadata stored alongside the actual float value within the PyFloatObject structure. By contrast, Seq infers the type of x at compile time and compiles the assignment to LLVM IR, which encodes type information. LLVM in turn compiles this to assembly or machine code.

Python has a relatively simple type system in which all types derive from the object base type. Some primitive types (such as integers and floats) are, for performance reasons, implemented directly in C within CPython’s runtime. However, even the C implementations of these types carry a significant overhead, as they still have to interoperate with the rest of the Python ecosystem. As can be seen in Figure 3, a simple float object—arguably the most lightweight type Python has—consists of three pointers, an integer and finally the float value itself. This “metadata” is necessary for Python’s runtime type resolution and reference counting (we do note, however, that the _ob_next and _ob_prev pointers are compiled into the structure definition conditionally, and can be omitted). For these reasons, all high-performance Python libraries (such as NumPy) achieve their speed by dealing primarily with arrays or matrices that can be abstracted away from the Python runtime to the C level.

Seq follows a different design philosophy in terms of types. Primitive types such as int, bool and float map directly to the equivalent LLVM IR types i64, i8 and double, respectively. As such, they incur no overhead whatsoever. Nevertheless, each of these primitives is still logically a fully-fledged type with a set of associated methods that can be extended by the user (e.g. type int has a method __add__ for addition that can be statically patched); there is no overhead as all method dispatches are resolved by the compiler. Furthermore, Seq inlines all magic method invocations on primitive types (e.g. an int.__add__ call is compiled to a single LLVM add instruction).

More complex types typically compile to an LLVM aggregate type or a pointer to one. Aggregate types are used in place of Python’s tuples and named tuples (represented in Seq by a new type construct), and are fully isomorphic to C structs. Pointers to aggregate types—or, more precisely, *reference types*—are used to implement classes (represented in Seq by a class construct). As usual, aggregates are passed by value while reference types are passed, unsurprisingly, by reference. For example, the following Seq expressions have types mapping to the indicated LLVM types:

**Table:** 

Seq expression	LLVM IR type	Description
“hello world”	{i64, i8*}	struct of length and character pointer
(1, 0.5, False)	{i64, double, i8}	struct of tuple element types
MyClass()	i8*	pointer to heap-allocated MyClass struct
MyClass().foo	{i8*, void (i8*)*}	struct of self and method function pointer

where the last example assumes foo is defined to be a method of MyClass that takes no extra arguments and does not return a value (i.e. def foo(self: MyClass) -> void).

In order to maintain compatibility with Python, class members can be deduced automatically by lexically analyzing a given class’s methods. Python’s built-in collection types—list, set and dict— are all modeled as reference types in Seq and bootstrapped as standard library classes implemented in Seq itself.

#### Generic Functions, Methods and Types.

Python’s lack of static typing allows any function to take objects of any type as an argument. This design philosophy does not translate well to strongly-typed compiled languages that lack runtime type information, as they typically require each function to explicitly specify input and output types.

Code compatibility with Python is of paramount importance for Seq, as it is unreasonable to expect users to manually annotate (or rewrite) their large codebases. Thus, Seq handles this problem by treating each Python function that does not provide type annotations as a *generic function*, where one or more input or output types cannot be deduced from annotations or a lexical analysis of the function body. In this case, each argument without a type annotation (referred to as an *implicit generic*) is replaced by a concrete type on demand at compile time. For example, on encountering f(42) as in Figure 5, the compiler checks whether there is an instantiation of f that accepts an int argument, and if so routes the call there. If not, the compiler clones f’s AST and creates a new instantiation of the function that specifically accepts an int argument (a similar approach is taken by Julia [Bezanson et al. 2012]). This newly created function would produce an error if, for example, int did not contain an appropriate __mul__ method as required in the function body. Instantiations are created lazily on demand.

**Fig. 4.**
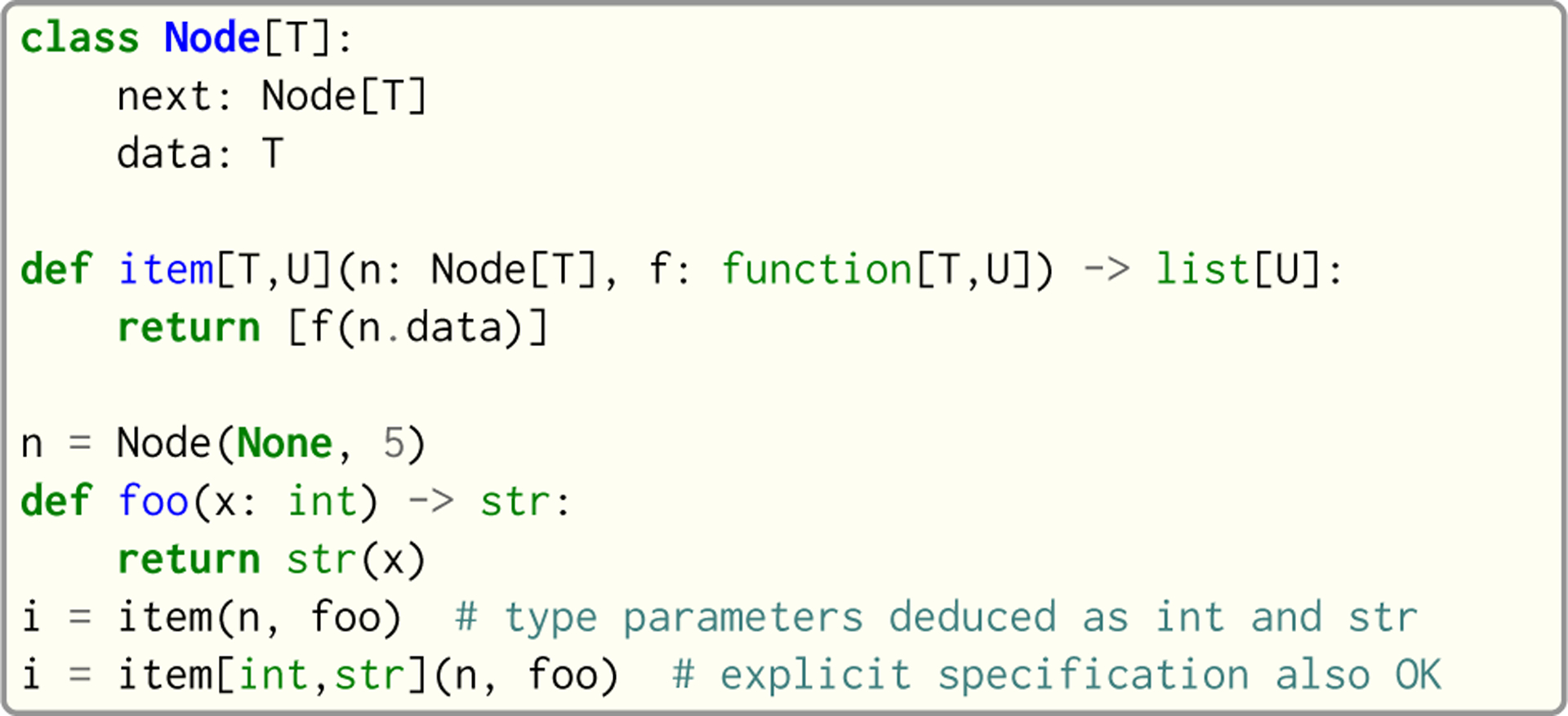
Seq’s explicit generic type parameters.

**Fig. 5.**
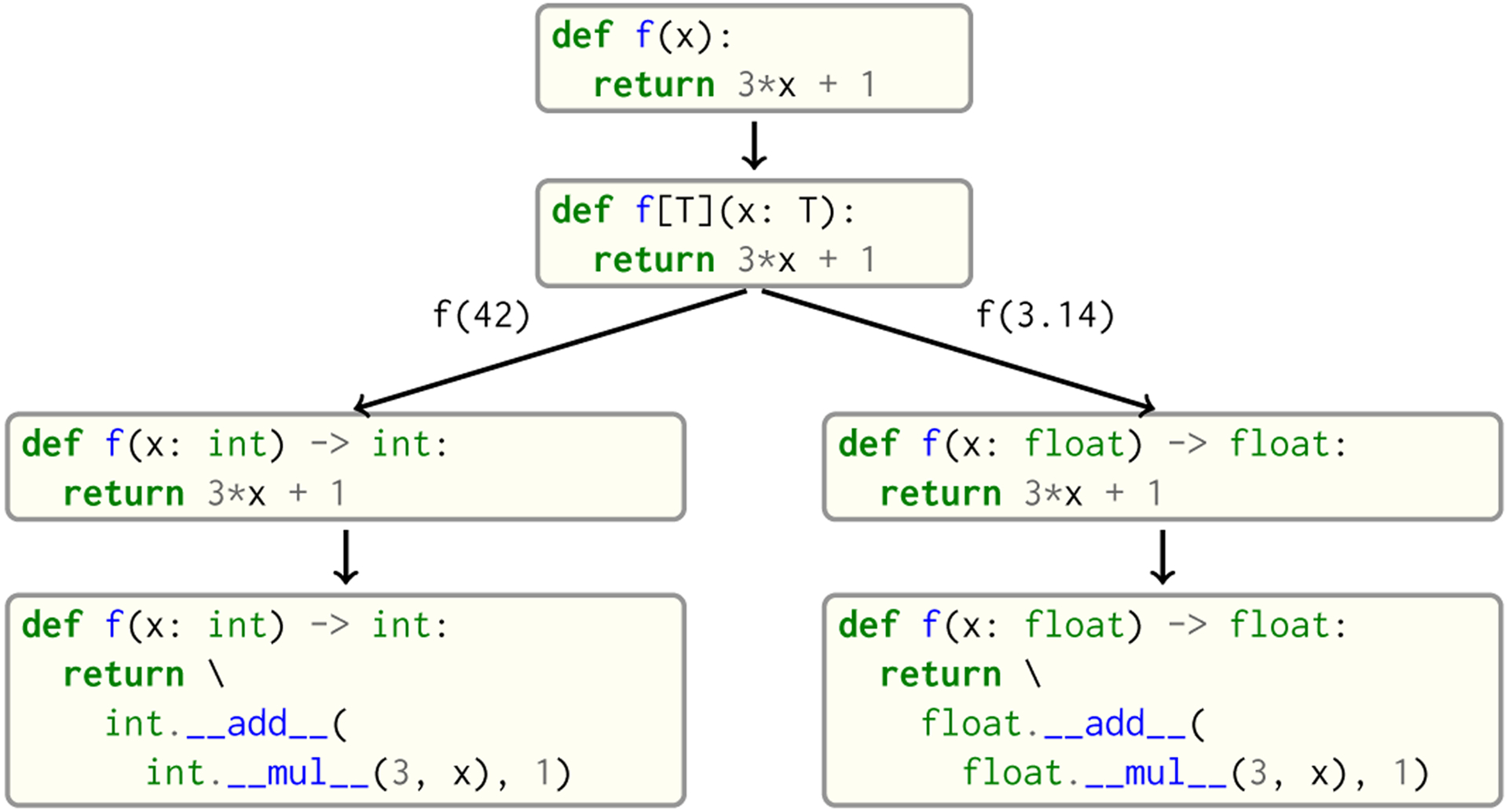
Seq’s implicit generic type parameters. The function f is declared to take a parameter x of unspecified type; the Seq compiler treats the type of x as generic and clones f on demand for each new input type, and subsequently deduces return types.

Unlike Python, Seq allows users to explicitly mark functions as generic and to specify explicit generic type parameters, allowing more complex type relationships to be expressed. For example, Figure 4 shows a higher-order function that only operates on generic nodes and functions (as T is an explicit generic type parameter). The argument types of item ensure that the argument function can take the argument node’s data as a parameter. Note that it is impossible for Python-style unnamed generics to cover this use-case without explicit isinstance checks. As shown in Figure 4, explicit type parameterization is optional even when explicit generics are present, as Seq performs type parameter inference whenever possible.

Analogous reasoning applies to classes, where class members can be generic. Examples of such classes include list[T] and dict[Key,Value]. Unlike functions, implicit generics are disallowed in classes as they would impair readability and could lead to ambiguous instantiations during the class member deduction stage. Note that, as far as Seq is concerned, different instantiations of functions and classes are treated as different types. Thus, f(x: list[int]) and f(x: list[float]) are represented internally as two separate functions, which allows Seq to optimize each instantiation according to its concrete argument types (albeit by sacrificing any kind of polymorphism, at least in the current implementation).

#### Duck Typing.

Seq’s type system is designed to behave like Python’s if one uses Seq as a drop-in Python replacement without specifying explicit types. As long as the methods of every type are known at compile time (an invariant strictly enforced by Seq as it does not allow type modifications at *runtime*), the compiler will deduce the argument/return types of all methods and instantiate any generic method as appropriate. Indeed, we find that this static instantiation-on-demand simulates duck typing reasonably well. Explicit type annotations enforce an extra layer of typing discipline on top of duck typing (à la mypy), and as such coexist peacefully with it.

#### Type Inference.

Any strongly typed language needs a way to infer the type of each variable present in a given program. Languages such as C or Pascal require end users to manually annotate each variable with a type. Other languages, such as C++11 or newer versions of Java, support uni-directional type inference by automatically deducing types of left-hand side terms based on right-hand side types. Initial versions of Seq also used uni-directional type inference, allowing users to say, for instance, x = 5 instead of x: int = 5.

However, uni-directional type inference is unable to handle a few common constructs in the Python language, including empty lists (e.g. a = []), nullables (e.g. a = None) and lambda functions (e.g. lambda x: x+1). With uni-directional inference, each of these constructs requires the user to provide manual type annotations (e.g. a: list[int] = []) even if the type can be inferred later. Because of this, Seq uses bi-directional type inference, implemented on top of the Hindley-Milner inference algorithm, to automatically annotate such types^2^. We slightly modified the standard Hindley-Milner algorithm to support generic classes, functions and instantiations on demand. We also enforce an invariant where all types within a scope (be it a function scope, class scope or the top-level scope) must be fully deduced by the end of that scope. This implies that a function cannot return a non-instantiated generic type: def f(): return [], for example, will cause a compilation error, but def f[T]() -> list[T]: return [] will compile successfully. Any weakly typed variable or lambda is instantiated as soon as possible (note that Seq treats lambdas as weakly typed constructs and does not generalize them—generalizations are only applied to generic functions defined with def and generic classes).

#### Limitations.

The strongly-typed nature of Seq does come with some limitations compared to conventional Python. Since all types must be fixed at compile time, a Seq program cannot (for example) create a collection of elements (e.g. list) with varying types. Seq’s tuples are also less versatile than Python’s: they cannot be iterated over if they contain different types, and a list cannot be cast to a tuple easily, as tuple sizes must be known at compile time. Seq also does not support method or class monkey-patching at runtime (but it does support this at compile time—see Type Extensions for details), nor indexing into a heterogeneous tuple with a non-constant index (as the type of the resulting expression would be ambiguous). Our type checker and instantiation algorithm also require each function to have a single return type. Finally, while Seq supports class extensions, it does not support subtyping (nor, therefore, fully-fledged polymorphism), meaning that class A; class B(A) will copy A’s methods to B without making B a subtype of A per se. With these trade-offs, Seq can perform all type-checking at compile time without sacrificing any runtime cycles for type enforcement, and without significantly hindering the expressibility of Python’s syntax. We have found that, especially in bioinformatics software, these language capabilities are seldom required (or at least can almost always be replaced by Seq-conforming alternatives with minimal effort); indeed, we are not aware of any genomics application that directly relies on such features. Consequently, these features are omitted in Seq at the time of writing. A brief list of differences between Seq and Python can be found in Appendix A.

### 4.2 Coroutines and Generators

Generators—Python’s answer to streams and lazy data structures—are an integral part of most Python/Seq programs: even simple for-loops are realized as an iteration over a generator. While it would certainly be possible to implement generators as they are in CPython (heap-allocated generator objects that expose a __next__ method for obtaining the next generated value), this would incur a substantial overhead, given how frequently generators are used.

**Fig. 6.**
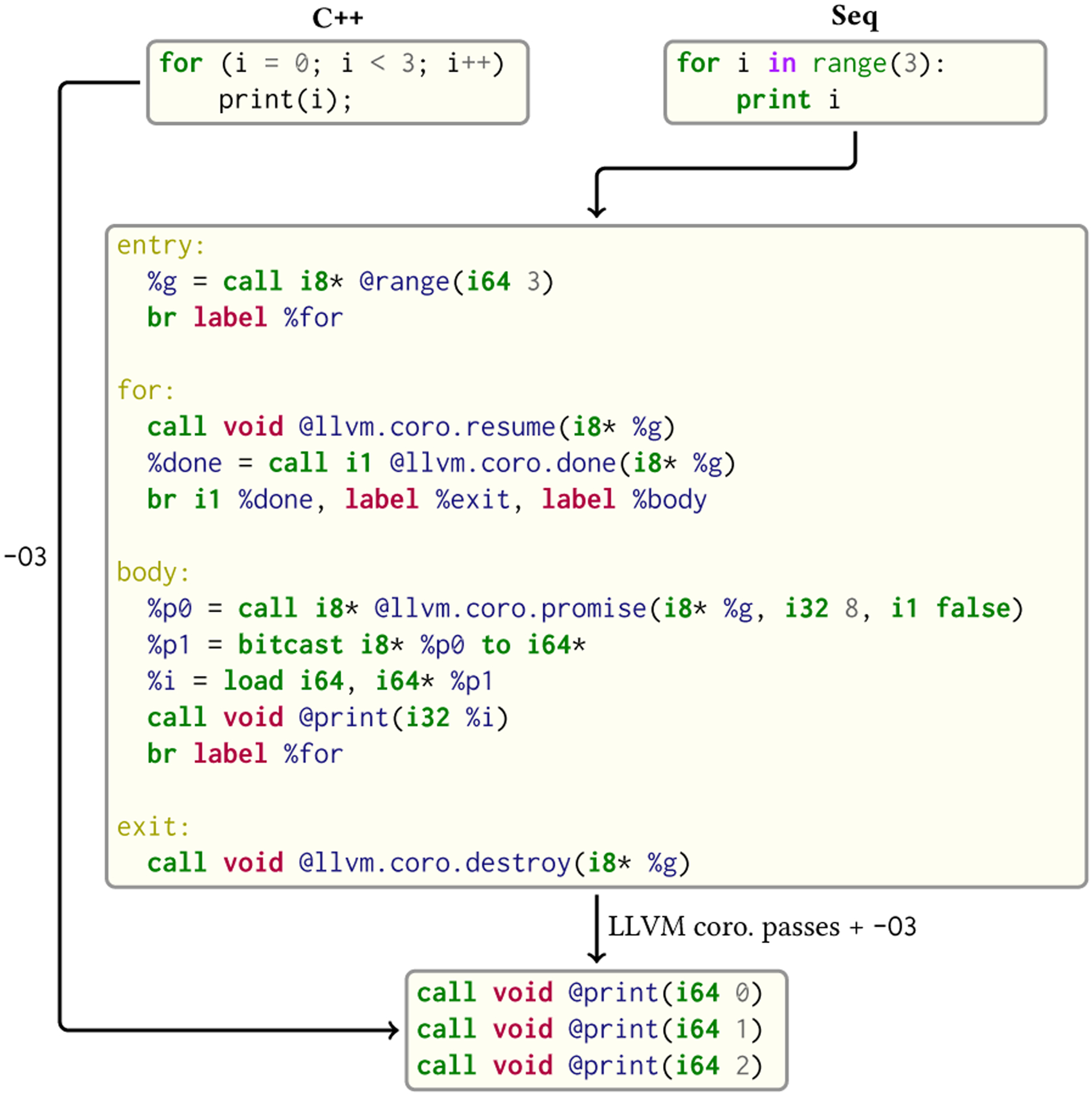
Compilation of Seq generators. Two semantically identical loops in C++ and Seq are shown in the uppermost boxes. Seq generators are implemented as LLVM coroutines, iteration over which in LLVM IR is shown in the middle box. The LLVM coroutine passes subsequently deduce that the “range” coroutine is created and destroyed in the same function without escaping, and inline/unroll the coroutine to produce code identical to the C++ example’s.

Instead, Seq employs LLVM coroutines (also used by Clang versions 6 and later to implement the C++ Coroutine TS [Nishanov 2017]). The advantage of this approach is that, when a generator is created and destroyed in the same function without escaping (by far the most common case in Python, similar to the for-loop example), LLVM’s coroutine passes are able to optimize out all the associated coroutine overhead, like coroutine frame allocation etc. Thereby, a typical Seq for-loop ultimately compiles to identical LLVM IR as the same loop expressed in C or C++, as shown in Figure 6.

### 4.3 Additional and Genomics-Specific Language Features

#### Sequence and k-mer Types.

Seq’s namesake type is indeed the sequence type: seq. A seq object represents a DNA sequence of any length and—on top of general-purpose string functionality—provides methods for performing common sequence operations such as splitting into subsequences, reverse complementation and *k*-mer extraction. Alongside the seq type are *k*-mer types, which are dependent on the *k*-mer length. For example, Kmer[1] represents a 1-mer, Kmer[2] a 2-mer and so on, up to Kmer[1024] (a reasonable upper bound on *k*-mer length in nearly any genomics application).

**Fig. 7.**
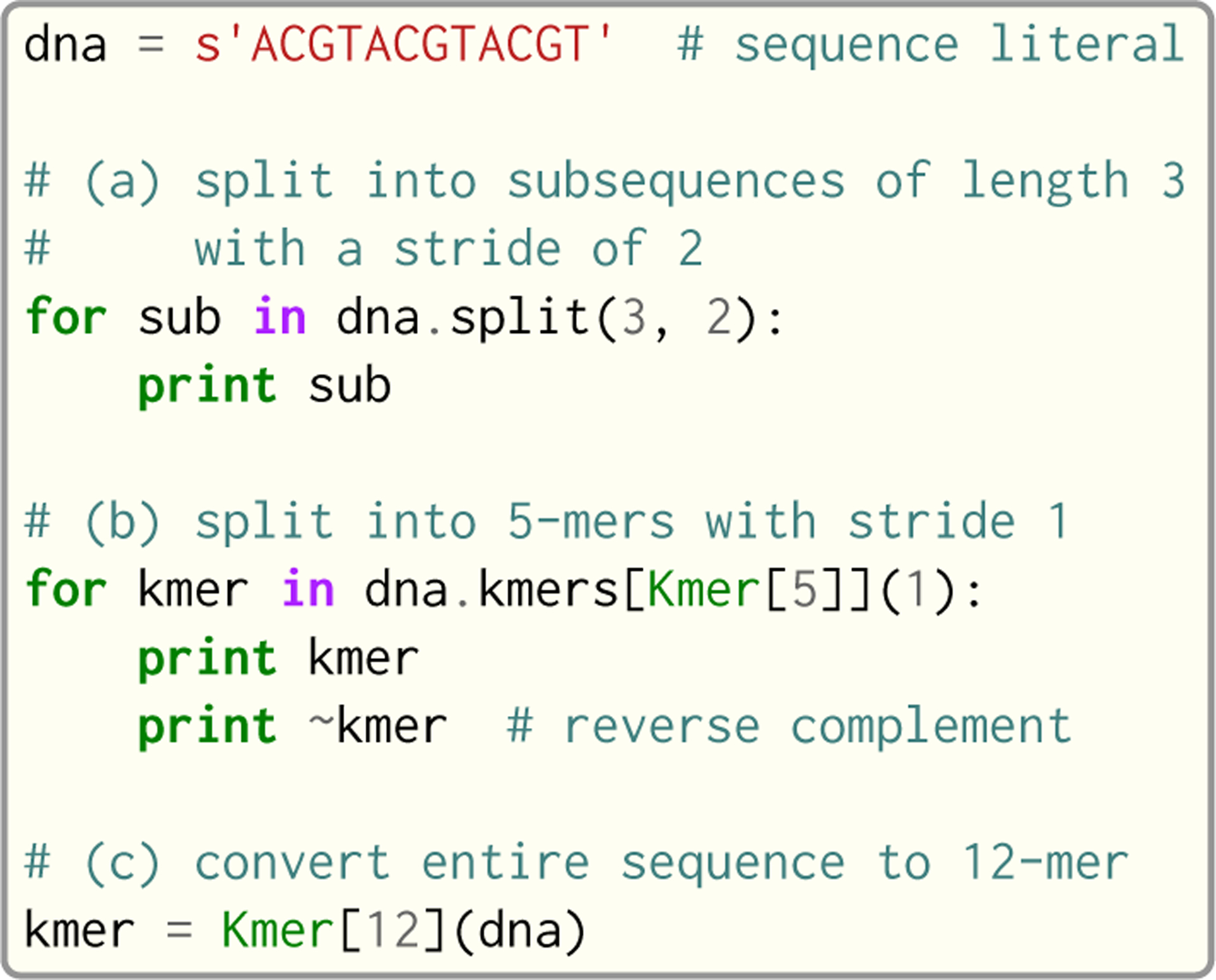
Example of seq and *k*-mer type usage.

Sequences can be seamlessly converted between these various types, as shown in Figure 7. In fact, this pattern is prevalent in many genomics applications, where longer sequences (be it a read, reference or anything else) are split into their constituent *k*-mers, and each is subsequently processed.

#### Pipelines and Partial Calls.

Pipelining is a natural model for thinking about processing genomic data, as sequences are typically processed in stages (e.g. read from input file → split into *k*-mers → query *k*-mers in index → perform full dynamic programming alignment → output results to file), and are almost always independent of one another as far as this processing is concerned. Because of this, Seq supports a pipe operator: |>, similar to F#’s pipe and R’s magrittr (%>%) [Bache and Wickham 2014]. Pipeline stages in Seq can be regular functions or generators. In the case of standard functions, the function is simply applied to the input data and the result is carried to the remainder of the pipeline, akin to F#’s functional piping. If, on the other hand, a stage is a generator, the values yielded by the generator are passed lazily to the remainder of the pipeline, which in many ways mirrors how piping is implemented in Bash. Note that Seq ensures that generator pipelines do not collect any data unless explicitly requested, thus allowing the processing of terabytes of data in a streaming fashion with no memory and minimal CPU overhead.

**Fig. 8.**
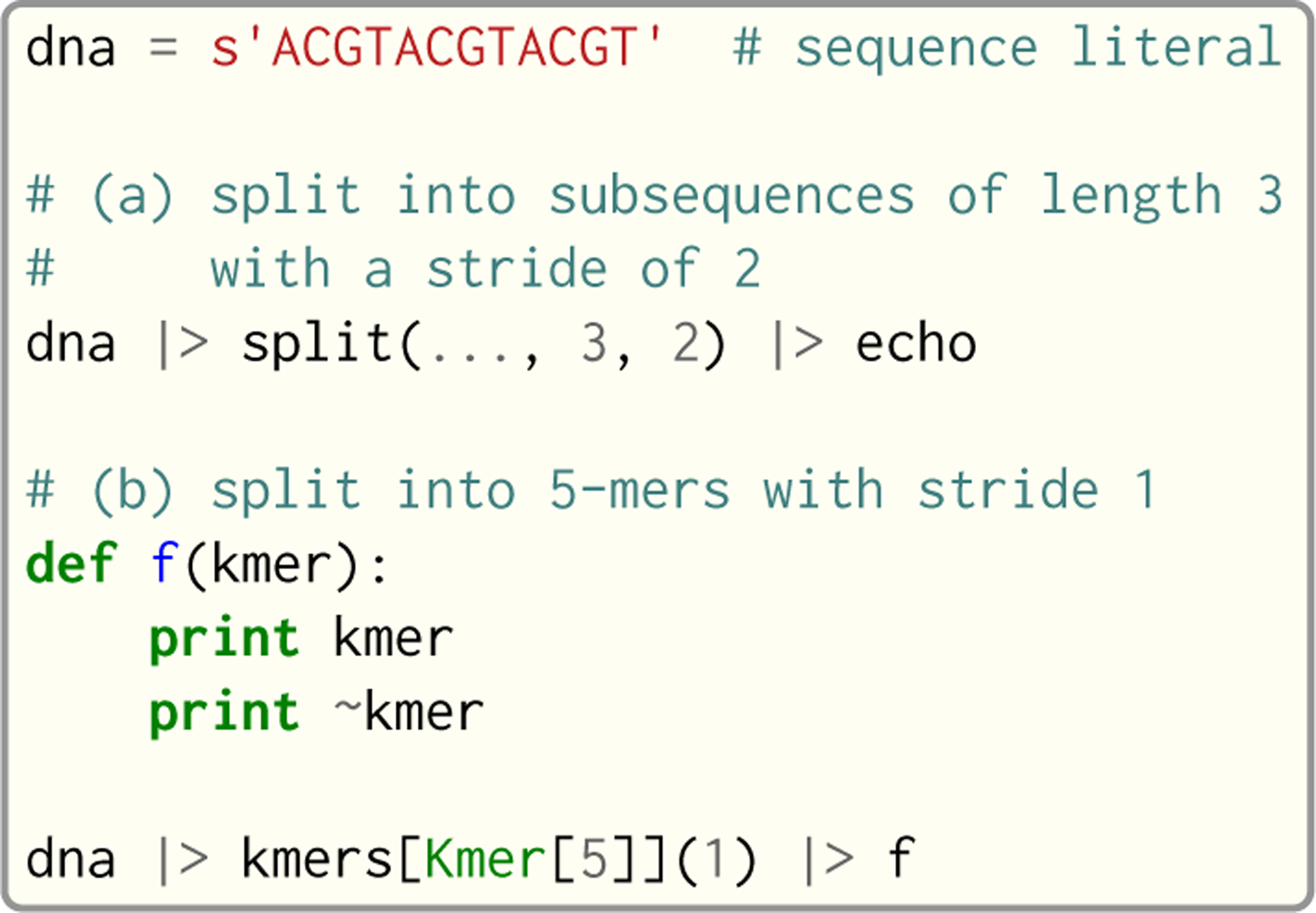
Example of pipeline usage in Seq, where the two loops from Figure 7 are represented as pipelines.

An example of pipeline usage is shown in Figure 8, which shows the same two loops from Figure 7, but as pipelines. First, note that split is a Seq standard library function that takes three arguments: the sequence to split, the subsequence length and the stride; split(…, 3, 2) is a partial call of split that produces a new single-argument function *f* where *f*(*x*) = split(*x*, 3, 2). The undefined argument(s) in a partial call can be implicit, as in the second example: kmers (also a standard library function) is a generic function parameterized by the target *k*-mer type and takes as arguments the sequence to *k*-merize and the stride; since just one of the two arguments is provided, the first is implicitly replaced by … to produce a partial call (i.e. the expression is equivalent to kmers[Kmer[5]](…, 1)). Both split and kmers are themselves generators that yield subsequences and *k*-mers respectively, which are passed sequentially to the last stage of the enclosing pipeline in the two examples.

#### Pattern Matching.

**Fig. 9.**
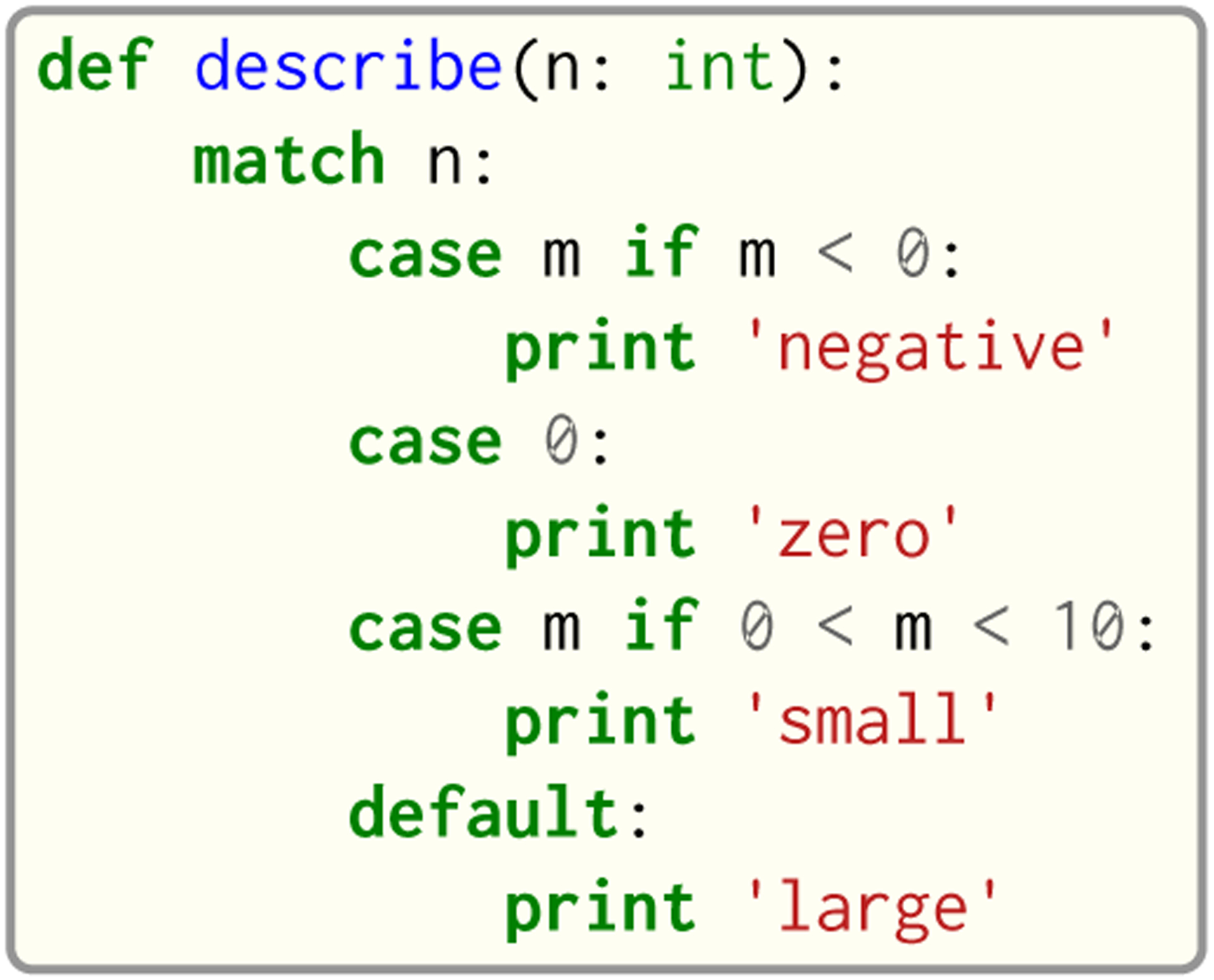
Example usage of match.

**Fig. 10.**
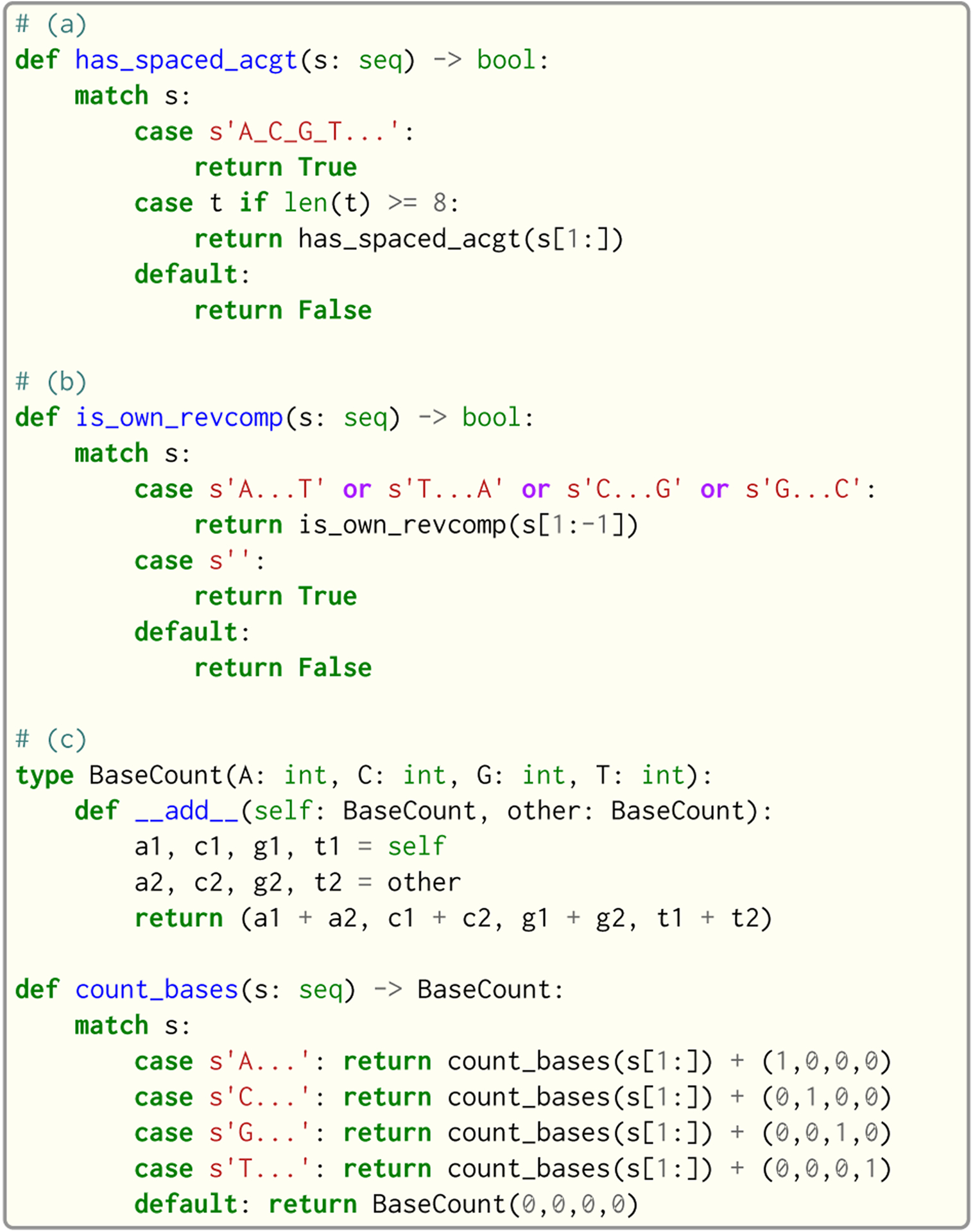
Example usages of match on sequences in Seq. Example (a) checks if a given sequence contains the subsequence A_C_G_T, where _ is a wildcard base; such an operation may be present in an application that uses spaced seeds—non-contiguous *k*-mers that are shown to improve accuracy in some settings [Kucherov et al. 2015]. Example (b) checks if the given sequence is its own reverse complement, which is useful in certain sequence hashing schemes [Ondov et al. 2016]. Finally, example (c) counts how many times each base appears in the given sequence, which can e.g. be used to determine GC content (the fraction of bases that are G or C) [Šmarda et al. 2014].

Seq provides the conventional match construct, which works on integers, lists, strings and tuples. An example usage of match is shown in Figure 9. A novel aspect of Seq’s match statement is that it also works on sequences, and allows for concise recursive representations of several sequence operations such as subsequence search, reverse complementation tests and base counting, which are shown in Figure 10. Sequence patterns consist of literal ACGT characters, single-base wildcards (_) or “zero or more” wildcards (…) that match zero or more of any base.

#### External Functions.

**Fig. 11.**
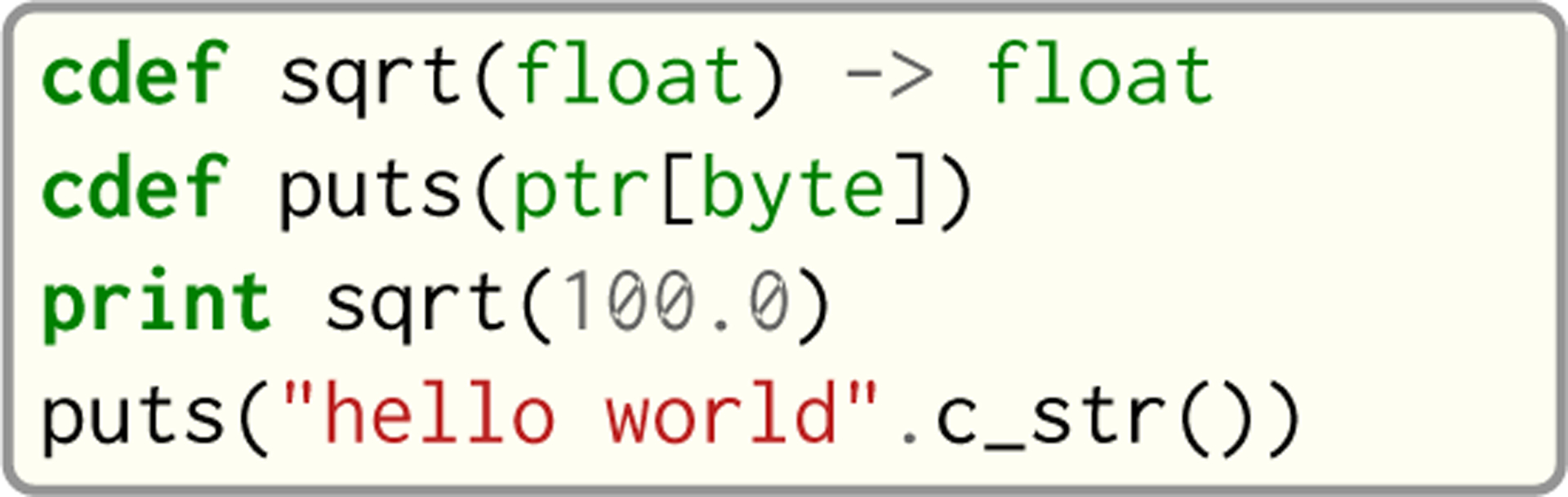
Example of cdef function usage in Seq with C standard library functions sqrt and puts.

Seq enables seamless interoperability with C and C++ via cdef functions, as shown in Figure 11. Primitive types like int, float, bool etc. are directly interoperable with the corresponding types in C/C++, while compound types like tuples are interoperable with the corresponding struct types. Other built-in types like str provide methods to convert to C analogs, such as c_str() as shown in Figure 11.

#### Type Extensions.

**Fig. 12.**
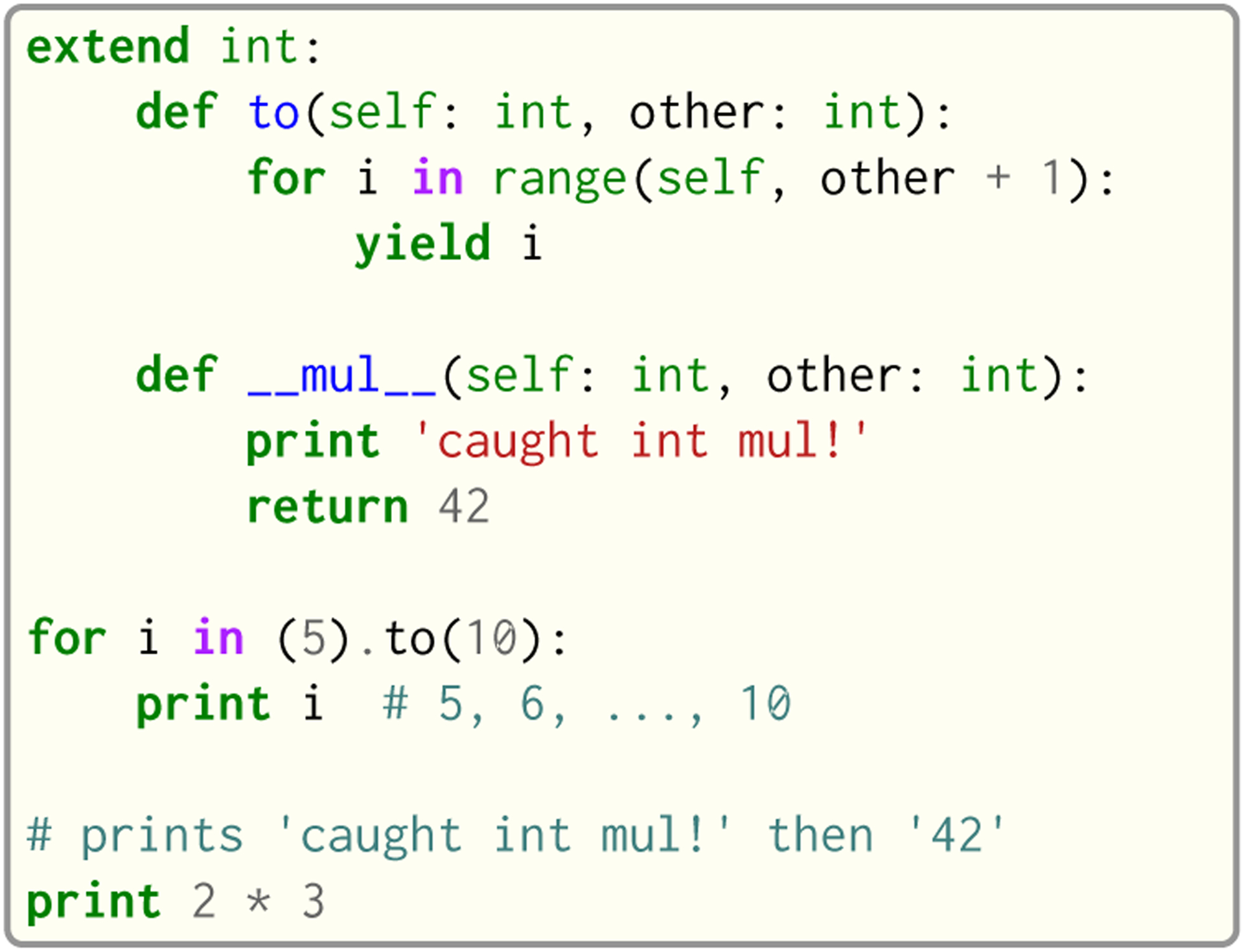
Example of type extension in Seq.

Seq provides an extend keyword that allows programmers to add and modify methods of various types within the current module at compile time, including built-in types like int or str. This allows much of the functionality of built-in types to be implemented in Seq as type extensions in the standard library. Figure 12 shows an example where the int type is extended to include a to method that generates integers in a specified range, as well as to override the __mul__ magic method to “intercept” integer multiplications. Note that all type extensions are performed strictly at compile time and incur no runtime overhead.

## 5 OPTIMIZATIONS

### 5.1 Sequence Encoding

The first and most straightforward optimization made by Seq is to 2-bit encode *k*-mer objects, as is commonly done in practice in performance-critical applications. In particular, we map *k*-mer types to the LLVM IR type iN where N = 2*k*. This has the advantage of allowing *k*-mers up to *k* = 32 to fit into a single machine word on 64-bit architectures.

In order to support fast *k*-mer operations, we also conditionally compile various lookup tables into any Seq program that requires them:

- For reverse complementation, we create a complete 4-mer reverse complement lookup table, implemented as a global length-4^4^ array indexed by encoded 4-mers, storing the encoded reverse complement of the given 4-mer at each index (hence, this array requires 256 bytes). Then, using the property $\bar{s_{1}\|s_{2}}=\bar{s_{2}}\|\bar{s_{1}}$ (where $\bar{s}$ denotes the reverse complement of a sequence *s* and ∥ denotes concatenation), we can construct the reverse complement of an arbitrary *k*-mer by partitioning it into 4-mers and concatenating (i.e. shifting and bitwise-ORing) their reverse complements in reverse, each of which is given by the lookup table. For *k* < 4, or the remainder of a longer *k*-mer whose length is not divisible by 4, we can simply pad with A-bases to obtain a 4-mer then remove the corresponding T-bases in the reverse complemented 4-mer (recall the reverse complement of A is T). Another noteworthy aspect of this scheme is that, if we choose our encoding wisely, we get reversal for free as well. Specifically, if we 2-bit encode base *b* ∈ {A, C, G, T} as *f* (*b*) so that *f* (A) = ~*f* (T) and *f* (C) = ~*f* (G) (where ~ is bitwise-NOT), then we can undo the “complementation” component of the reverse complement to obtain the original sequence in reverse by applying a simple bit inversion.
- For converting general sequences into *k*-mers, we compile a second lookup table that maps the ASCII characters A, C, G and T to their 2-bit encoded values, implemented also as a length-256 array. The encoding process then iteratively looks up each base in this array to construct the encoded *k*-mer.
- For converting *k*-mers back to sequences, we use a simple length-4 array that maps the 2-bit encodings back to ASCII. We note, however, that this is a far less common conversion than the previous one.

### 5.2 Parallelism

**Fig. 13.**
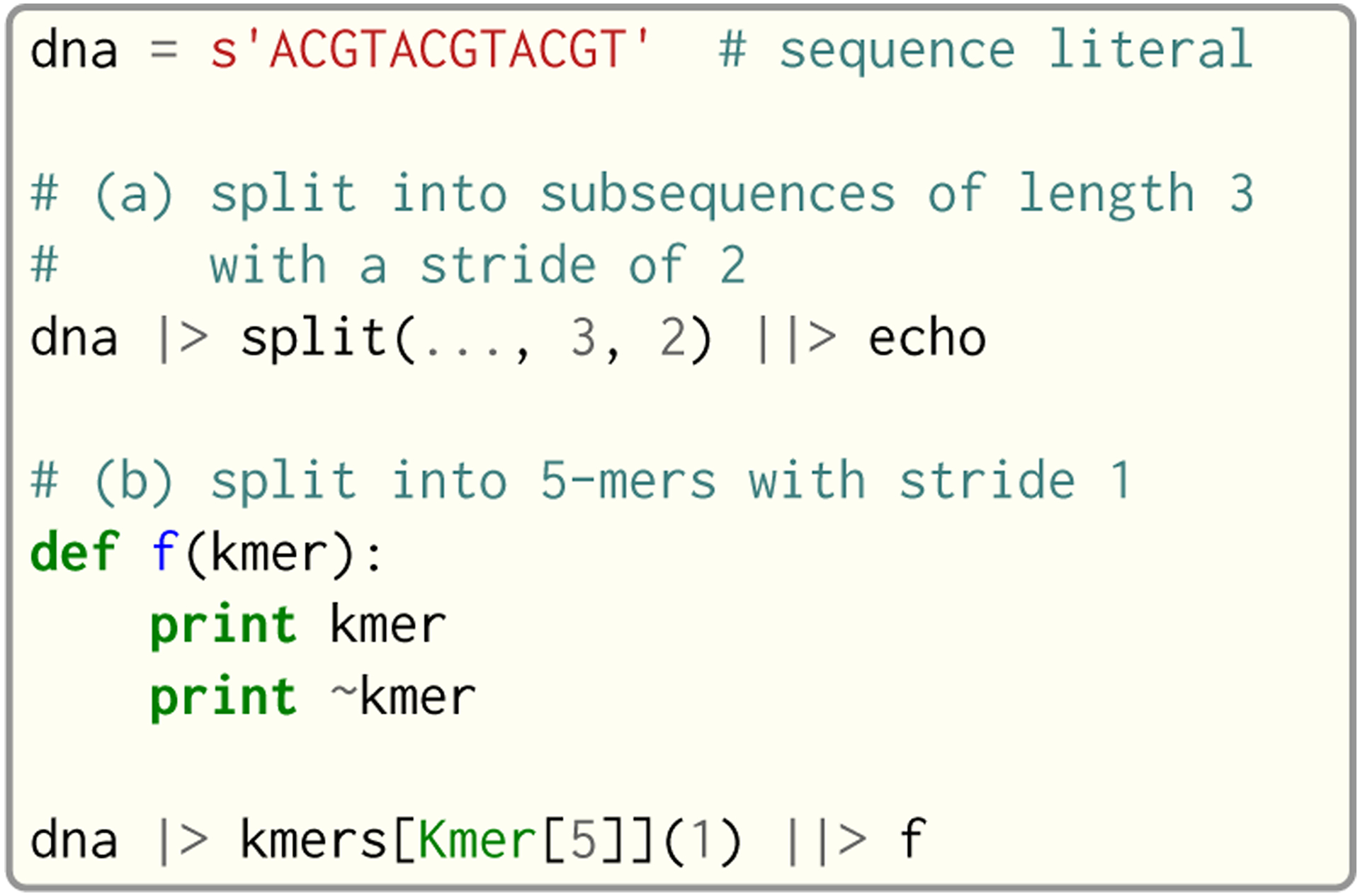
Example of parallel pipeline usage in Seq, where the two pipelines from Figure 8 are parallelized.

CPython and many other implementations alike cannot take advantage of parallelism due to the infamous global interpreter lock, a mutex that protects accesses to Python objects, preventing multiple threads from executing Python bytecode at once. Unlike CPython, Seq has no such restriction and supports full multithreading. To this end, Seq supports a *parallel* pipe operator ||>, which is semantically similar to the standard pipe operator except that it allows the elements sent through it to be processed in parallel by the remainder of the pipeline. Hence, turning a serial program into a parallel one often requires the addition of just a single character in Seq, as shown by Figure 13. Further, a single pipeline can contain multiple parallel pipes, resulting in nested parallelism.

Internally, the Seq compiler uses Tapir [Schardl et al. 2017] with an OpenMP task backend to generate code for parallel pipelines. Logically, parallel pipe operators are similar to parallel-for loops: the portion of the pipeline after the parallel pipe is extracted into a new function that is called by the OpenMP runtime task spawning routines (as in #pragma omp task in C++), and a synchronization point (#pragma omp taskwait) is added after the outlined segment. Lastly, the entire program is implicitly placed in an OpenMP parallel region (#pragma omp parallel) that is guarded by a “single” directive (#pragma omp single) so that the serial portions are still executed by one thread (this is required by OpenMP as tasks must be bound to an enclosing parallel region).

### 5.3 Software Prefetching for Faster Genomic Index Lookups

Large genomic indices—ranging from several to tens or even hundreds of gigabytes—used in many applications in the field result in extremely poor cache performance and, ultimately, a substantial fraction of stalled memory-bound cycles [Appuswamy et al. 2018; Wang et al. 2012; Zhang et al. 2007]. For this reason, Seq performs pipeline optimizations to enable data prefetching and to hide memory latencies, an idea that has also been explored in previous work [Chen et al. 2007; Kiriansky et al. 2018]. The programmer must provide just:

- a __prefetch__ magic method definition in the index class, which is logically similar to __getitem__ (indexing construct) but performs a prefetch instead of actually loading the requested value (and can simply delegate to __prefetch__ methods of built-in types);
- a one-line prefetch hint indicating where a software prefetch should be performed, which can typically be just before the actual load.

For instance, an index that consists of a single array v may implement __getitem__(self,x) by returning self.v[x], in which case it would implement __prefetch__(self,x) by returning self.v.__prefetch__(x); i.e. the prefetch is delegated to the underlying array (which may in turn be delegated to a raw pointer, which has intrinsic methods for *actually* performing the prefetch via an LLVM prefetch instruction).

**Fig. 14.**
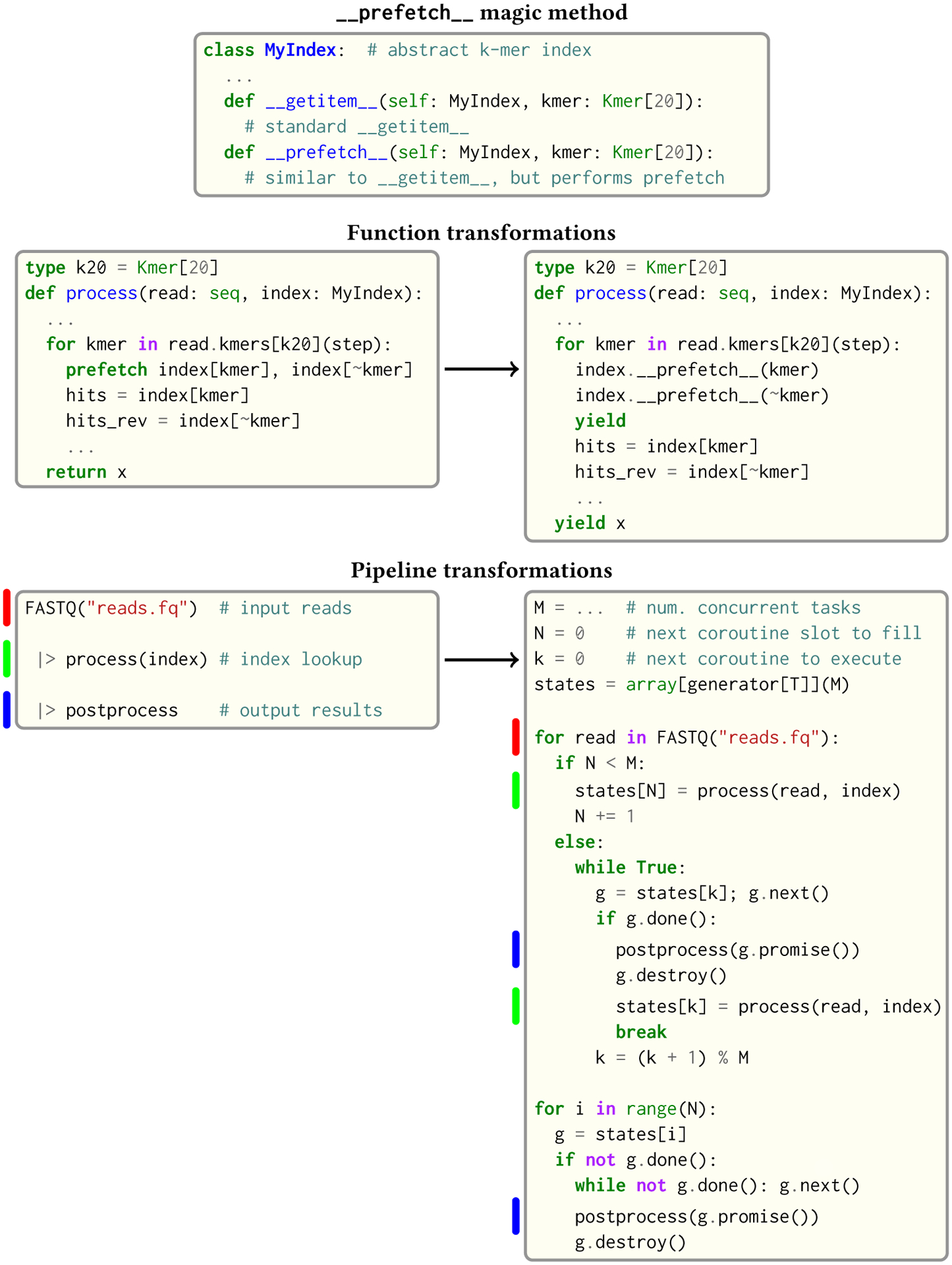
Transformations performed by Seq to enable effective index prefetching. Colored segments under pipeline transformations indicate where the specific stages show up in the resulting code. FASTQ is the standard file format for storing sequencing reads.

A full example is shown in Figure 14. First, prefetch statements themselves compile to explicit invocations of the __prefetch__ method, and functions containing prefetch statements (such as process in the figure) are converted by the compiler into coroutines that yield after each prefetch. Then, pipelines containing such functions as stages are transformed into loops that dynamically schedule multiple invocations of the newly created coroutine, where once one invocation yields or terminates, another is resumed or created by the scheduler, respectively. The transformed pipeline in Figure 14, for example, has several noteworthy components:

- M is the number of concurrent coroutines to be processed, which ideally should be large enough to saturate the memory bandwidth of the processor as prefetches are performed. In practice, we choose M conservatively to be 16, which also allows for software prefetching performed by other parts of the system, such as the garbage collector.
- N is a variable indicating how many of the M coroutine slots have been filled, and is only used at the start of the loop to actually fill the slots.
- k is the next coroutine slot to be resumed by the loop.
- states is the array holding the M coroutine handles/frames (which have type generator in Seq). In reality this array is stack-allocated in the entry block of the function containing the pipeline. (T is simply the original return type of process.)

The code generated in the loop body is that of a simple dynamic scheduler where:

- The if N < M component initially populates the array of pending coroutines states.
- Inside the else clause is a loop that iterates cyclically through states and resumes each coroutine. If a coroutine terminates (i.e. if g.done()), then the value returned by the coroutine (given by g.promise()) is sent through the remainder of the pipeline, as it would be in the original untransformed pipeline; then, the coroutine is destroyed and a new one is created to take its place.
- The final loop simply completes any remaining coroutines that have not yet terminated. Since the number of such coroutines is at most M, this loop just executes them sequentially.

By employing this scheme, the latency of one coroutine’s cache miss can be overlapped with useful work from another, increasing memory-level parallelism and overall throughput. Note that these optimizations depend only on the existence of a prefetch instruction, which is the case for nearly any modern architecture.

## 6 EVALUATION

We evaluated the performance of Seq on the following three benchmark suites, designed to mimic both hypothetical and real-world genomics applications:

1. *The Computer Language Benchmarks Game* suite [Gouy [n. d.]] restricted to DNA benchmarks (3 benchmarks)
2. Sequence manipulation suite, developed in-house (3 benchmarks)
3. Genomic index queries (2 benchmarks)

We compared Seq with C++ (compiled with both GCC v7.4.0 and Clang v6.0.1), Julia v1.0.3, Python v2.7.15, PyPy v2.7.13 [Bolz et al. 2009], Shed Skin v0.9.4 [Dufour 2006] and Nuitka v0.6.2 [Hayen 2012]. Other “compiled Python” implementations such as Numba are geared towards numerical rather string or DNA processing, and had issues efficiently compiling our benchmarks, or were abandoned. All experiments were run on a dual-socket system with Intel Xeon X5690 CPUs (3.46 GHz) with 6 cores each (totalling 12 cores and 24 hyper-threads) and 138GB DDR3–1333 RAM with 12MB LLC per socket. C++ implementations were compiled with −03 -march=native. Julia was run with --chec*k*-bounds=no −03 parameters. Shed Skin was run with -l -o optimizations, and Nuitka was run with the noasserts, no_warnings options enabled. Note that Seq binaries (unlike C++ or Julia) do include bounds checks.

For the Benchmarks Game suite, we used the FASTA, RevComp and *k*-nucleotide microbench-marks. Other benchmarks in this suite are not directly relevant to genomics or bioinformatics in general, but we expect Seq’s performance on them to be on par with that of other LLVM-backed languages. Briefly, the FASTA benchmark entails generating random sequences in the FASTA format; RevComp entails reverse complementing a set of longer sequences, which is done through Seq’s domain-specific sequence type; *k*-nucleotide entails counting *k*-mers of various lengths, which we implemented using Seq’s *k*-mer types.

For the in-house suite, we designed three microbenchmarks that capture common genomics operations on a large set of reads:

1. RC: Output the reverse complement of each read, also implemented using sequence types. Unlike RevComp, this benchmark runs on millions of shorter reads rather than a few long reads.
2. 16-mer: Count the number of symmetric 16-mers in all reads (where a 16-mer is symmetric if its first half is identical to the reverse complement of its second half). The Seq implementation for this benchmark uses match on sequences, and is based on example (b) in Figure 10.
3. CpG: Count the number of CpG regions (i.e. regions that consist of C and G characters, such as CGC but *not* CCC or GGG as they lack a G and C, respectively) in all reads, and report the lengths of the shortest and longest CpG regions in the sample.

The final benchmark suite demonstrates the utility of Seq’s domain-specific genomic index query optimizations. Here, we use the genomic indices implemented in the widely-used tools SNAP v1.0 (beta 23) [Zaharia et al. 2011] (a sequence alignment tool) and SGA v0.10.15 [Simpson and Durbin 2012] (a *de novo* assembly tool). SNAP uses a hash table of 20-mers, which we re-implemented from scratch in Seq (see Appendix B). SGA, on the other hand, uses an FM-index, whose C++ implementation we wrapped in Seq. Both indices are used to query *k*-mers from our test dataset. For both SNAP and SGA, we compared the performance of our base Seq implementation, a Seq version that performs pipeline optimizations for index prefetching (code difference of one line) and a C++ implementation (results shown at the bottom of Table 2). Both of these benchmarks consume roughly 30GB of RAM.

**Fig. 15.**
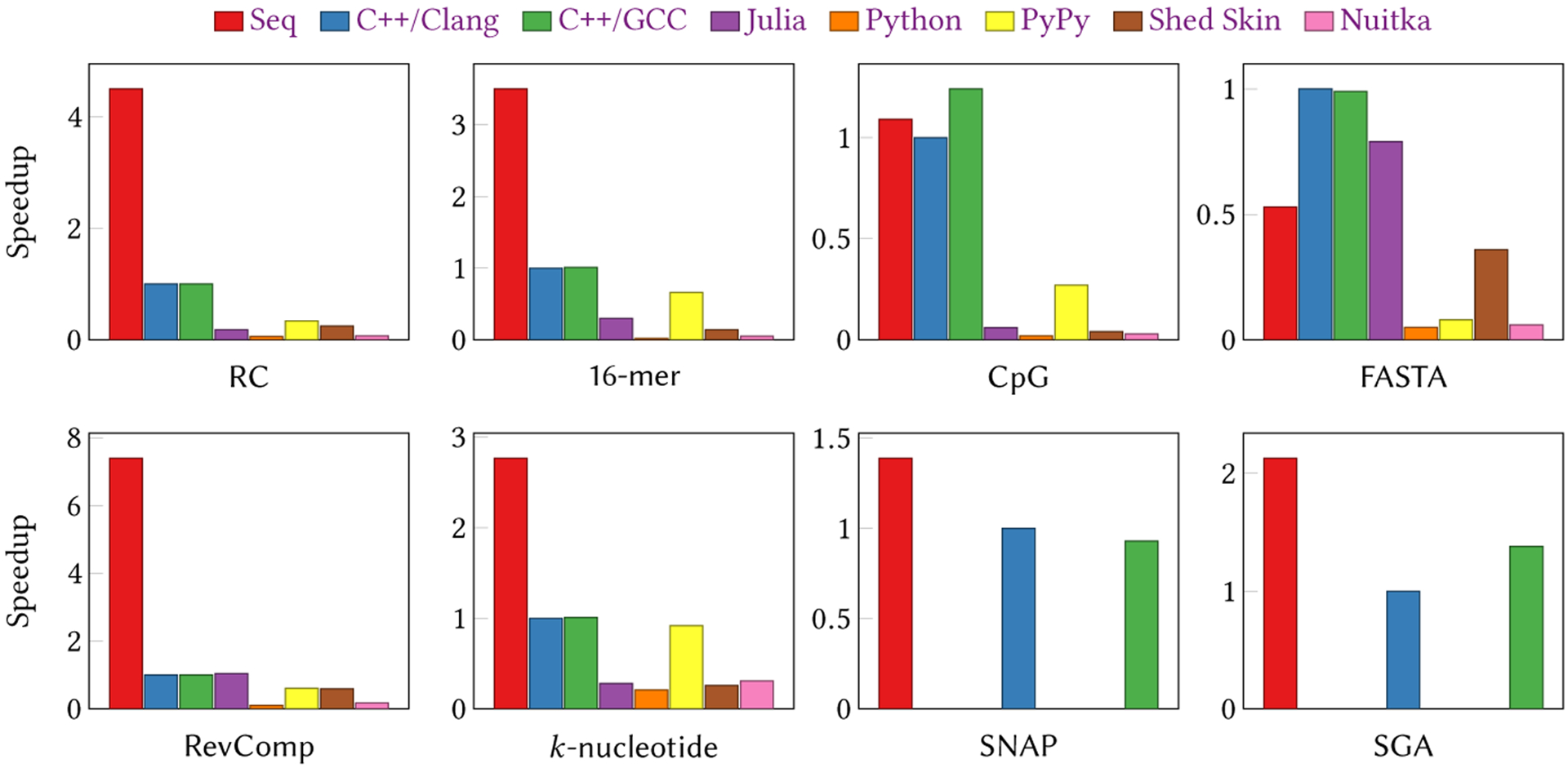
Seq evaluation results on several genomics benchmarks, showing speedups over Clang. The Seq implementations used in these charts use Seq-specific types and constructs that are not available in Python. Note that only Seq, Clang and GCC were tested on the SNAP and SGA benchmarks. Seq performs at least as good as (and in many cases much better than) the C++ implementations in nearly every benchmark, excluding FASTA which, as we note in the text, is not as common a real-world application as the others.

Each benchmark was executed five times for each language/compiler, and the averages are reported (with the exception of Julia, Python, Shed Skin, PyPy and Nuitka, which were run three times as they took orders of magnitude longer in some cases). The input dataset consisted of 100 million 75bp DNA reads randomly chosen from the HG00123 sample [1000 Genomes Project Consortium et al. 2010] (because SGA’s index is an order of magnitude slower than SNAP’s, we downsampled our input dataset to 25 million reads for SGA). Results are shown in Figure 15, where speedups over Clang-compiled C++ are given.

### 6.1 Improvements over Python

Seq programs can be written in one of two ways: in plain Python or using idiomatic Seq (as in the implementations described above). The Pythonic implementations embody the conventions set by the Python community, and code written in this way can be easily run by both Python and Seq without modifications. The alternative style involves the use of idiomatic Seq constructs and data types to manipulate genomic data, which are not available in plain Python.

We compare the performance of Pythonic and idiomatic Seq implementations to that of Python, PyPy, Shed Skin, Nuitka and Julia in Table 1. All of the implementations in the second benchmark suite (with the exception of idiomatic Seq) are line-by-line identical in terms of the algorithm. In particular, the Python implementations use the exact same code as the Pythonic Seq implementations for RC, CpG and 16-mer. Even by just directly running Python code, Seq is able to outperform Python by a factor of 11 to 100.

A similar pattern can be seen in the first benchmark suite, where Seq significantly outperforms both the Python and Julia implementations. The only exception is the FASTA benchmark, where Seq is slightly slower than Julia. While this could be further optimized, we chose to keep the version that is most similar to Python. Additionally, we note that the FASTA benchmark as specified by the Computer Language Benchmarks Game is not a realistic application in genomics, as one would rarely be generating sequences rather than reading them from a preexisting dataset.

Idiomatic versions further boost the improvement up to 160×, and showcase the impact of individual domain-specific optimizations: RC and RevComp utilize Seq’s highly optimized reverse complementation constructs; 16-mer showcases the gains—both in terms of readability and performance—of sequence-based match statements; *k*-nucleotide shows the performance improvement gained by using Seq’s native *k*-mer types. A few of the idiomatic versions also rely on pipelining to perform further optimizations, which is described in more detail below.

Note that runtime becomes prohibitive as the number of reads to be processed increases; while the performance of compiled Python (i.e. PyPy, Shed Skin and Nuitka) and Julia is acceptable if the number of reads is low (as in the first benchmark suite), it rapidly deteriorates once the read count becomes an order of magnitude larger. Even 100 million reads as used here is quite minuscule compared to real datasets.

Given the results above, the comparisons below focus only on the C++ implementations.

### 6.2 Improvements over C++

Table 2 compares the Seq and C++ implementations of each benchmark. Again, all of these implementations (with the exception of idiomatic Seq) are line-by-line identical in terms of the algorithm. The performance of Pythonic Seq code is on par with that of C++ code—in most cases, it is the same as or slightly better than Clang’s (we use Clang as a baseline since both Clang and Seq rely on LLVM for general-purpose optimizations). Note that g++ is sometimes able to outperform the LLVM-based backends of Seq and Clang. However, once Seq applies domain-specific optimizations, it outperforms even g++ by up to 4×. For example, in the third set of real-world benchmarks (SNAP and SGA), Seq achieves a 50% speedup after adding a one-line domain-specific prefetch statement to the original code, resulting in up to a 2× speedup over C++.

#### Prefetch Variability.

Index prefetching is useful during genomic index lookups, and is able to speed up both *k*-mer hash tables and FM-indices by 50%. However, we observed the performance of prefetching to be application- and data-dependent: while in almost all evaluated datasets (spanning various technologies such as recent third-generation 10x Genomics linked-reads [Zheng et al. 2016] and “classic” second-generation Illumina short-reads; detailed results omitted for brevity) it produces a steady improvement in the range 20–50%, in one dataset it led to a 35% slowdown. However, the fact that it is a one-line change means that any user can easily experiment and judge whether it works well for their use-case.

#### Compilation Time.

Seq can be used in two modes: as a JIT interpreter or as a compiler. In our experiments, Seq’s compilation times are faster than, or similar to, those of the C++ compilers. Note that Seq relies on LLVM’s optimization pipeline, and therefore employs the same optimization passes (and linker) as Clang. We also observed that Seq is an order of magnitude faster than Nuitka or Shed Skin with respect to compilation.

#### Manual Optimizations.

In general, it is not straightforward to compare benchmark results across different languages in a meaningful way, given that each language has its own set of idioms and conventions, often resulting in algorithmic differences. For example, if one invests enough time, it is always possible to write C++ implementations that match Seq’s performance, since both ultimately compile to machine code. For this reason, the in-house benchmarks above all follow the same high-level algorithm, even if there may be room for further manual optimizations. The other two sets of benchmarks do include hand-optimized C++ implementations, however: FASTA, RevComp and *k*-nucleotide implementations are taken from The Computer Language Benchmarks Game (excluding multithreaded implementations), and SNAP and SGA are real-world implementations that are widely used in practice.

We additionally compared to the highly-optimized bioinformatics libraries SeqAn v2.3.2 [Döring et al. 2008] (C++), BioPython v1.74 [Cock et al. 2009] and BioJulia (BioSequences v1.1.0) (https://biojulia.net), results for which are shown in Table 3. Seq outperforms both BioPython and BioJulia by a large margin. Seq also substantially outperforms SeqAn on RC; on CpG, the plain C++ implementation actually outperforms both; lastly, SeqAn outperforms Seq on 16-mer. Note that Seq matches SeqAn’s performance if the same low-level implementation is used (the SeqAn implementation differs and is less flexible because Seq’s sequence pattern matching cannot be expressed in SeqAn/C++). However, we purposefully compared against the (somewhat slower) pattern matching Seq implementation to show that even the “simplest” implementations in Seq are close performance-wise to the highly-optimized implementations provided by other libraries. Finally, these libraries are unable to easily express the benchmarks from the two other suites (and also lack a prefetching mechanism similar to what Seq uses in SNAP and SGA), which is why we limited this comparison to the in-house benchmarks.

### 6.3 Effects of Parallelization

To evaluate the performance of Seq’s parallel pipelines, we implemented parallel versions of two of our in-house benchmarks, CpG and 16-mer (the third, RC, performs substantially more I/O and hence does benefit much from parallelism), as well as SGA’s FM-index querying (both with and without prefetch optimizations). To this end, we “block” input reads into batches of 100,000, which are processed as a whole by each task; tasks themselves are then executed in parallel via Seq’s parallel pipe operator.

Results are shown in Table 4, where Seq scales almost linearly up to 4 threads on our in-house benchmarks. For these small applications, we find I/O to be a bottleneck beyond 4 threads. In a real-world setting where reads would take substantially longer to process, we would expect I/O to play a less significant role, allowing a greater degree of parallelism. Taking these parallel implementations into account, Seq’s largest speedup over Python (which cannot be easily parallelized due to the global interpreter lock) is over 650×. Finally, Table 4 also shows that even Seq’s prefetching optimizations benefit from parallelization.

## 7 RELATED WORK

A few methods have been proposed to aid in the development of bioinformatics tools and workflows. One approach focuses on building specialized libraries for genomic data manipulation. Examples include C++ libraries such as SeqAn [Döring et al. 2008] and htslib [Li et al. 2009b], and high-level libraries such as BioPerl and BioPython [Cock et al. 2009]. However, none of these libraries solve the aforementioned problems, as none can both scale with the size of NGS data and allow for sufficiently high-level representations of bioinformatics or genomics algorithms. Additionally, the use of libraries prevents optimizations like operator fusion, or the pipeline transformations made by Seq. Another line of work focuses on integrating various high-level code blocks into a pipeline framework that can be efficiently run on large clusters and cloud-based systems—examples include the Broad Institute’s HAIL project and Workflow Description Language [Voss et al. 2017]. While these methods indeed allow large-scale parallelism and a relatively high-level description of a given problem, they are cumbersome to use as they require rather expensive infrastructural setup and administration costs, and do not tackle the problem of single-machine optimizations, which is still a significant bottleneck in many pipelines.

The DSL proposed in this paper is inspired by many successful DSLs that already exist in other fields of computer science [Abadi et al. 2016; Baghdadi et al. 2019; Chafi et al. 2011; Chiw et al. 2012; Kjolstad et al. 2017, 2016; Ragan-Kelley et al. 2013; Zhang et al. 2018]. Despite their substantial success in these other areas, computational biology has yet to adopt a comparable DSL. SARVAVID [Mahadik et al. 2016] is a DSL designed for computational genomics applications, which provides a set of high-level genomics kernels and exposes them as language constructs. For example, common operations such as *k*-merization, index-generation, index-lookup, similarity-computation and clustering are provided. While such an approach provides efficient implementations of these kernels and combinations thereof, it lacks generality, which is gravely needed in the field as new sequencing technologies produce new types of data that in turn necessitate novel algorithms. Seq aims to provide a more general, lower-level language, with general-purpose constructs that can be used to build a variety of kernels efficiently. While there also exist a few general-purpose languages optimized for scientific computing such as Julia [Bezanson et al. 2012] and MATLAB, neither of these languages is designed for computational biology workflows.

On the Python side, recent Python standards introduced type hints, which allow static type checking [van Rossum 2015]. Projects such as Cython [Behnel et al. 2011], PyPy [Bolz et al. 2009], Numba [Lam et al. 2015], Shed Skin [Dufour 2006] and Nuitka [Hayen 2012] all aim to generate efficient code by relying on ideas such as static type checking and (JIT-) compiling rather than interpreting Python. While Seq is similar to these language in that it is indeed compiled and uses static type checking, Seq also uses domain-specific information to apply further code optimizations, and introduces data types that are tailored to the field of computational biology. Furthermore, many of these other implementations still rely on the Python runtime, and are thus bound to its inherent performance overhead. For the sake of completeness, a comprehensive comparison between Seq and other Python implementations is given in Table 5. In this work, we chose to compare to PyPy, Shed Skin and Nuitka primarily because other similar implementations (e.g. Numba, Pythran, Pyston, Grumpy) are either geared more towards scientific/numerical computing or no longer under active development.

## 8 CONCLUSION

We have introduced Seq, a new language for computational biology that offers the productivity of Python and the performance of C. Thereby, Seq bridges the gap between computationalists who seek to write performance-critical code for a particular application, and biologists whose day-to-day workflow involves rapid development and prototyping of new ideas and algorithms. Through Seq, we introduce several novel genomics-specific language constructs and optimizations, which collectively attain a 160× performance improvement over standard Python, and up to 7× improvement over C++. Future work includes exploring a wider range of domain-specific optimizations that exploit the unique structure of biological data.

## Figures and Tables

**Table 1.** Seq runtime compared to Python, PyPy, Shed Skin, Nuitka and Julia (seconds). “Seq (Py.)” (Pythonic Seq) uses the same code as Python, whereas “Seq (Id.)” (idiomatic Seq) uses Seq-specific language features and constructs.

	Seq (Py.)	Python	PyPy	Shed Skin	Nuitka	Julia	Seq (Id.)	Speedup
FASTA		111.3	68.6	15.6	99.4	**7.1**	10.7	0.7–10×
RevComp		97.5	15.5	16.0	54.3	9.1	**1.3**	7–75×
*k*-nucleotide		255.8	59.2	211.8	174.1	196.0	**19.7**	10–13×
RC	195.1	2,160.0	231.2	912.1.6	913.7	764.7	**48.3**	5–45×
CpG	60.6	3,591.0	203.7	1,336.9	1,596.4	947.7	**60.6**	16–60×
16-mer	159.8	15,440.2	463.4	2,139.9	6,042.3	1,030.9	**95.6**	5–161×

**Table 2.** Seq runtime compared to C++ as compiled with Clang and GCC (seconds). “Pythonic Seq” uses the same code as Python, whereas “Idiomatic Seq” uses Seq-specific language features and constructs. For SNAP and SGA the difference between the without-prefetch and with-prefetch Seq programs is just a single prefetch statement.

	Seq	C++	C++	Seq	Speedup
	Pythonic	Clang	GCC	Idiomatic	
FASTA		5.6	5.7	10.7	0.5×
RevComp		9.5	9.5	**1.3**	7.3×
*k*-nucleotide		54.6	54.3	**19.7**	2.8×
RC	195.1	178.6	170.7	**48.3**	3.5×
CpG	60.6	55.7	**44.8**	60.6	0.7–0.9×
16-mer	159.8	214.1	201.7	**95.6**	2.1×
	Seq	C++	C++	Seq	Speedup
	w/o prefetch	Clang	GCC	with prefetch	
SNAP	328.1	450.5	327.5	**211.9**	1.5–2.1×
SGA	453.0	569.3	610.1	**409.6**	1.4–1.5×

**Table 3.** Seq runtime compared to that of several highly-optimized bioinformatics libraries (in seconds) on in-house benchmarks. C++ times are also included for reference.

	Seq	C++	SeqAn	BioPython	BioJulia
		GCC	C++/GCC	PyPy	Julia
RC	**48.3**	170.7	137.6	68.4	348.9
CpG	60.6	**44.8**	46.7	332.7	244.1
16-mer	95.6	201.7	**70.8**	1,276.1	247.2

**Table 4.** Seq runtimes on multiple threads (seconds).

Threads	1	2	3	4	Speedup
CpG	58.1	29.6	19.9	15.3	3.8×
16-mer	86.7	43.6	29.9	22.8	3.8×
SGA	355.1	184.0	125.4	95.1	3.7×
SGA (pref.)	217.7	128.0	90.3	71.8	3.0×

**Table 5.** Comparison between Seq and other Python implementations. For “Domain”, “Bio.” means computational biology, “Sci.” means scientific computing and “Astro.” means astrophysical computing. “Unknown types” refer to types that cannot be statically determined. Also note that Pyston has several JIT tiers in addition to its LLVM JIT.

	Domain	Target	Compilation	Unknown types allowed?	Full Python?	CPython runtime?	Multithreading?
**CPython**	General	Bytecode	Interpreted	✓	✓	✓	✗
**Seq**	Bio.	LLVM IR	AOT	✗	✗	✗	✓
**Cython**	General	C	AOT	✗	✓	✓	✓
**PyPy**	General	Bytecode	Interpreted	✓	✓	✓	✗
**Numba**	Sci.	LLVM IR	JIT	✓	✗	✓	✗
**Nuitka**	General	C++	AOT	✓	✓	✓	✗
**Pythran**	Sci.	C++	AOT	✓	✓	✗	✓
**Pyston**	General	LLVM IR	JIT	✓	✓	✓	✗
**HOPE**	Astro.	C++	JIT	✗	✗	✗	✗
**Shed Skin**	General	C++	AOT	✗	✗	✗	✓
**Grumpy**	General	Go	AOT	✗	✗	✗	✓

## Box

CCS Concepts: • **Applied computing** → **Computational genomics; Bioinformatics**; • **Software and its engineering** → **Domain specific languages**.

## Footnotes

1
http://seq.csail.mit.edu

2 This is a recent addition to Seq, and is currently still in the testing stage. At the time of writing, Seq’s master branch uses uni-directional type deduction with added support for generics and type-less nullables. Note that this uni-directional version does not support lambdas.

## A SEQ VS. PYTHON – A CHEAT SHEET

### A.1 Additional Types

- seq: Represents a genomic sequence.
- Kmer[*N*] (1 ≤ *N* ≤ 1024): Represents a *k*-mer of length *N*. *N* must be constant.
- Int[*N*] (1 ≤ *N* ≤ 2048): Represents a signed *N*-bit integer (standard int is an Int[64]). *N* must be constant. The common type definitions i8, i16, i32 and i64 are provided in the standard library for convenience.
- UInt[*N*] (1 ≤ *N* ≤ 2048): Represents an unsigned *N*-bit integer. *N* must be constant. The common type definitions u8, u16, u32 and u64 are provided in the standard library for convenience.
- ptr[T]: Represents a pointer to type T; primarily useful for C interoperability.
- array[T]: Represents an array of type T (essentially a pointer with a length).

### A.2 Additional Keywords

- type: Declares a named tuple or a type alias.
- match/case/default: Match statement
- extend: Adds the given methods to an existing type.
- cdef: Declares an externally-defined C function.
- prefetch: Prefetches the given items in the given index objects.

Seq also provides __ptr__ for obtaining a pointer to a variable, and __array__ for declaring stac*k*-allocated fixed-size arrays.

### A.3 Static Types

Because Seq is statically-typed, lists (for example) cannot contain elements of different types as they can in Python. Similarly, a variable cannot be assigned to objects of different types, nor can a function return objects of different types. Seq currently also does not support polymorphism.

### A.4 Tuples

Tuples in Seq are implemented as structures. Consequently, heterogeneous tuples can only be indexed by a constant value, since otherwise the type of the index expression would be ambiguous. Similarly, iteration over a tuple is only possible if it is homogeneous.

### A.5 Scopes

Seq enforces slightly stricter variable scoping rules than standard Python. In short, a variable cannot be first assigned in a block then used afterwards for the first time in the enclosing block. This restriction avoids the problem of unitialized variables.

## B CODE FROM SNAP BENCHMARK

SNAP uses a hierarchical hash table as its genomic index. To index *k*-mers (*k* ≥ 16), an array of 4^*k*−16^ quadratic probing hash tables is created, indexed by every possible length-(*k* − 16) prefix. Each constituent hash table is then a mapping of 16-mer to genomic loci at which that 16-mer appears. To handle multiple loci for a single *k*-mer, an auxiliary array is used, and hash table values can be pointers into this array (determined by whether the value is greater than the largest locus). This hierarchical structure exploits the fact that not every length-(*k* − 16) prefix appears in the genome with equal frequency, so the size of each internal hash table can be chosen based on the corresponding prefix’s frequency.

SNAP’s index is implemented in C++, and can be found at https://github.com/amplab/snap. Below, we give the Seq implementation used in the SNAP benchmark. The code that queries this table largely resembles what is shown in Figure 2 (both for Seq and C++). Note that loading the precomputed table from disk is still done in C++, and wrapped in Seq.
